# Augmented Marshmallow Extract Lipid Nanoparticles with Clove Oil Embedded in Collagen Sponge for Ultimate Antimicrobial Healing of Diabetic Mouth Ulcer

**DOI:** 10.3390/pharmaceutics17050611

**Published:** 2025-05-05

**Authors:** Sammar Fathy Elhabal, Ahmed Mohsen Faheem, Sandra Hababeh, Jakline Nelson, Nahla A. Elzohairy, Yasmine F. Ibrahim, Tassneim M. Ewedah, Ibrahim S. Mousa, Khaled M. Allam, Ahmed Mohsen Elsaid Hamdan

**Affiliations:** 1 Department of Pharmaceutics and Industrial Pharmacy, Faculty of Pharmacy, Modern University for Technology and Information (MTI), Mokattam, Cairo 11571, Egypt; 2 Department of Medical Biochemistry and Molecular Biology, Faculty of Medicine, Mansoura University, Mansoura 35516, Egypt; dr_ahmed1986@yahoo.com; 3 Department of Pharmaceutics, College of Pharmacy, King Saud University, Riyadh 11451, Saudi Arabia; sandra.hababeh@outlook.sa; 4 Department of Microbiology and Immunology, Faculty of Pharmacy, Nahda University, Beni-Suef (NUB), Beni-Suef 62511, Egypt; jaklinekeka@gmail.com; 5 Department of Microbiology and Immunology, Faculty of Pharmacy, Modern University for Technology and Information (MTI), Mokattam, Cairo 11571, Egypt; nahla.elzohairy@pharm.mti.edu.eg; 6 Air Force Specialized Hospital, Cairo 19448, Egypt; 7 Department of Medical Pharmacology, Faculty of Medicine, Minia University, El-Minia 61511, Egypt; yfibrahim@fcms.edu.sa; 8 Pathological Sciences Department-MBBS Program, Fakeeh College for Medical Sciences, Jeddah 21461, Saudi Arabia; 9 Pharmaceutics and Pharmaceutical Technology Department, Faculty of Pharmacy, Egyptian Russian University, Cairo 11829, Egypt; tassneim-mohammed@eru.edu.eg; 10 Pharmaceutics Department, Faculty of Pharmacy, Sinai University, Al-Arish 45511, Egypt; ibrahim.salah@su.edu.eg; 11 Department of Pharmacognosy, Faculty of Pharmacy, South Valley University, Qena 83523, Egypt; khaled.mohamed@svu.edu.eg; 12 Department of Pharmacy Practice, Faculty of Pharmacy, University of Tabuk, Tabuk 71491, Saudi Arabia

**Keywords:** *Althaea officinalis*, anti-inflammatory, clove oil, collagen sponge, diabetic mouth ulcers, solid lipid nanoparticles, oral mucosal drug delivery, *Pseudomonas aeruginosa*, *Escherichia coli*, *Candida albicans*

## Abstract

**Background/Objectives**: Diabetic mouth ulcers are a pathological condition of the oral mucosa leading to increases in susceptibility to infection and prolonged wound healing time. Still, there is a lack of natural formulations for treating this condition. Our principal objective was to formulate solid lipid nanoparticles (SLNs) that contained *Althaea officinalis* (marshmallow) (M.) extract with clove oil (CO.), subsequently integrated into a collagen sponge for enhancing stability, solubility, sustained release, antimicrobial efficacy, and healing power when targeting diabetic oral ulcers. **Methods**: A factorial design of 34 trials was established to evaluate the influence of lipid concentration (A), SAA concentration (B), lipid type (C), and SAA type (D). The optimized M-CO-SLNs was selected using Design Expert^®^, the based Poly dispersibility index (Y2), zeta potential (MV) (Y3), and encapsulation efficiency (%) (Y4). The optimized SLNs were integrated into a collagen sponge matrix and tested for their antibacterial and antifungal efficacy against *Pseudomonas aeruginosa*, *Escherichia coli*, and *Candida albicans*, respectively. Moreover, they were tested for their wound healing power in a diabetic mouth ulcer model. **Results**: The optimized formula (Run 16: 5% lipid concentration, 4% SAA concentration, capric acid) demonstrated P.S (110 ± 0.76 nm), ZP (−24 ± 0.32 mV), PDI (0.18 ± 0.05), and EE% (90 ± 0.65%.). The optimized M-CO-SLNs formula was incorporated into a cross-linked collagen sponge and showed superior antimicrobial efficacy, an increased swelling ratio, and was effective in an in vivo oral ulcer study, as evidenced by ELISA biomarkers, gene expression analysis, and histological analysis. **Conclusions**: M-CO-SLNs embedded in collagen sponges is a promising therapeutic formula for clinical application against diabetic mouth ulcers.

## 1. Introduction

Oral mouth ulcers, recurring erosions or lesions in the oral epithelium, are globally very common. It has been reported that over 27% of the world’s population has mucosal injuries. Untreated ulcers can cause epithelial cell loss, leading to cavities or necrosis [1]. Speaking, eating, swallowing, and digesting may be difficult for the affected population. The therapeutic care plan for diabetic oral ulceration has some current problems and is debatable. The bacteria invading oral ulcerations form biofilms, leading to emerging resistance to most antibiotics [2]. In the same way, the production of a high level of reactive oxygen species (ROS) in these oral ulcers causes oxidative stress and tissue damage. Most therapeutic agents for diabetic wounds do not address impaired angiogenesis, which elongates the time needed for nutrients and oxygen to enter the wound [3]. Normal ointments and patches, even ones made with chitosan, do not always help diabetics when treating infections or combating oxidative stress and tissue regeneration. Because of these problems, we need diabetic oral sore treatments that can do more than one thing, in order to heal, especially in complicated conditions [4]. Several complex factors influence how diabetic patients’ oral ulcers heal. Chronic exposure to an inflammatory pathological environment significantly slows down the healing process. Active inflammatory cells produce high levels of pro-inflammatory cytokines such as TNF-α, IL-1β, and IL-6. Cytokines accumulate and remain at the wound site, causing significant damage [5]. Diabetic oral mucosal wounds heal slowly due to oxidative stress. An imbalance between ROS and antioxidants indicates oxidative stress. High glucose levels at the wound site cause mitochondria to produce high ROS levels, which causes mitochondrial dysfunction and interferes with VEGF binding to receptor 2 [6]. These disruptions impede wound healing by inhibiting endothelial cell migration and angiogenesis.

Diabetic mouth ulceration management starts with blood sugar control, since high glucose impairs ulceration, immunity, and wound healing [7]. Tight glycemic control reduces the frequency, severity, and healing time of oral ulceration. Topically applied lidocaine, benzocaine, and fluocinonide reduce pain and inflammation [8]. Chlorhexidine gels or rinses help diabetics with oral ulcers to avoid infections. Using these medications under medical supervision is essential in order to avoid poor prognosis and complications. Dental hygiene is important for diabetics because they are more susceptible to infection [9]. Dentists suggest antiseptic mouthwash to clean ulcers and prevent complications. Avoiding ulceration by eating bland, soft foods is also highly recommended. Hydration for softening oral ulcers is also highly recommended. Moreover, oral corticosteroids may be utilized to manage particularly severe ulcers [10]. These medications promote ulcer healing through a reduction in inflammation. Immunosuppressive or antiviral medications may alleviate severe autoimmune or infectious ulcers [11]. Natural extracts exhibiting anti-inflammatory, antimicrobial, and wound-healing properties may complement conventional treatments for diabetic oral ulcers. Many extracts contain bioactive compounds that aid recovery and complement conventional treatments [12]. Aloe Vera gel soothes and heals ulcers, and its antimicrobial and anti-inflammatory anthraquinones and polysaccharides speed up tissue repair and prevent infection [13,14]. The high viscosity of antibacterial honey, especially manuka, moisturizes tissue, protects ulcers, reduces pain, and speeds up healing. Honey’s anti-inflammatory properties speed up diabetic ulcer healing [15,16]. Anti-inflammatory, antioxidant-rich turmeric reduces diabetic oral ulcer inflammation and speeds up healing in the form of an ulcer paste or mouthwash [17]. Chamomile calms inflammation, and certain teas reduce ulcer pain and inflammation; inflammation can be lowered by the polyphenols found in green tea [18].

Roots and leaves of *Althaea officinalis*, known as marshmallow (M.), contain anti-inflammatory, antimicrobial, antioxidant, and soothing mucilage, flavonoids, and phenolic acids. Marshmallow can treat oral ulcers, sore throats, gastrointestinal issues, and skin wounds [3]. In addition, it is a mucilaginous plant that benefits mucous membranes, tissue regeneration, and infection prevention. Moreover, its antimicrobial properties aid those with weakened immune systems by healing ulcers and preventing secondary infections [19]. Marshmallow contains antioxidants, which reduce oxidative stress and improve health. In addition to this, clove oil and coconut oil can help treat oral ulcers by killing germs and reducing inflammation [20]. Directly applying these natural medicines to oral ulcers has been proven to reduce pain and swelling and help prevent infections. They are used traditionally as antiseptic and anti-inflammatory medicines, and neem (*Azadirachta indica*) mouthwash also cures ulcers [21].

Clove oil (CO.), derived from *Syzygium aromaticum* tree buds, has been widely used for centuries, owing to its aroma, flavor, and therapeutic properties [22]. Clove oil is packed with eugenol, which is known for its effective properties. It can help with pain relief, fight off germs, tackle fungi, reduce inflammation, and even act as an antioxidant [20]. This makes it useful for dealing with mouth pain, skin issues, and various oral problems like toothaches and gum inflammation. Eugenol relieves and numbs pain, treating gingivitis and periodontitis through impeding harmful bacteria. Clove oil has some interesting antimicrobial properties that people use to treat fungal infections, such as athlete’s foot, eczema, and acne [17]. Massage oils with antioxidants can really help with sore muscles and joints. They neutralize free radicals, reducing oxidative stress and improving health. Clove oil reduces inflammation, helping arthritis, muscle pain, and swelling. It may slow down aging, prevent cancer, and heart disease [23]. Clove oil is not just for applying to the skin; it also helps with digestion and breathing as well. It helps to ease bloating, nausea, and indigestion with some gentle carminatives. Clove oil is great for aromatherapy. It helps to clear mucus and can be used to treat coughs, colds, and even asthma [8].

Marshmallow and clove oil exhibit potential synergistic pharmacological effects; however, their therapeutic efficacy is constrained by poor aqueous solubility, low bioavailability, and sensitivity to pH, temperature, and light. Therefore, an innovative therapeutic strategy is required to address these limitations and enhance their ulcer-healing efficacy [24]. Advanced drug delivery systems were subsequently investigated. Because of their special characteristics, nanoparticles provide nanotechnology with hope for the discovery of new natural ulcer-healing treatments. Our focus has been on creating promising entities that can overcome traditional natural product solubility, stability, and toxicity for intended delivery [25]. By encapsulating hydrophobic and hydrophilic drugs, solid lipid nanoparticles (SLNs) enable active compound solubilization and regulated drug release. Confirming their stability, nanostructured lipid carriers, a modified generation of SLNs, encapsulate an oily phase of liquid lipids in a solid matrix to generate a structured network that supports drug solubility, non-toxicity, biocompatibility, and biodegradability [26]. SLNs incorporated into collagen sponges produce a homogeneous, biocompatible delivery matrix that enhances ulcer coverage and drug release. Proven collagen scaffold material preserves fibroblast shape and arrangement and stimulates cell proliferation for quicker ulcer healing, and also stimulates tissue development [27].

This study aims to create and characterize an innovative transmucosal oral ulcer drug delivery system utilizing solid lipid nanoparticles (M-CO-SLNs) infused with marshmallow and clove oil, incorporated into collagen sponges, employing Design Expert^®^ software (version 13). Natural clove oils, along with biodegradable and biocompatible materials, possess qualities that aid in the healing of oral ulcers. The great surface area of the SLNs and their proximity to the oral mucosa should help the absorption of marshmallow to improve through the oral mucosa. The proposed method incorporates M-CO into SLNs, which are then embedded into collagen-based sponges, thereby offering a promising delivery platform that addresses the limitations of conventional formulations. The justification for employing collagen sponges is based on their biocompatibility and porous structure. These sponges provide mechanical support, firmly adhere to the mouth ulcer site, and reduce the frequency of dressing changes, all of which are necessary for effective mouth ulcer management. When combined with a collagen scaffold, SLNs improve drug penetration, allow for controlled release, and produce a synergistic effect. Therefore, this study conducts in vitro, antimicrobial, and in vivo evaluations to investigate the mouth-ulcer-healing potential of the proposed M-CO-SLN sponges, providing a foundation for future clinical applications.

## 2. Materials and Methods

### 2.1. Materials

Stearic acid, capric acid, linoleic acid, and streptozotocin (STZ) were obtained from Sigma-Aldrich (St. Louis, MO, USA), with the following purities: stearic acid (≥99%), capric acid (≥99%), linoleic acid (≥99%), and streptozotocin (≥98%). Span 60, poloxamer 407, and poloxamer 188 (BDH Chemical Ltd., Poole, England) with purities of ≥99% were used. Clove oil was purchased from Sigma-Aldrich (CAS No. 8000-34-8), Mumbai, India. *Althaea officinalis* leaves were obtained from the Haraz Company (Cairo, Egypt). Collagen from bovine Achilles tendon, methanol, and acetonitrile (HPLC grade) was obtained from Fluka Chemika-BioChemika, Buchs, Switzerland. Toll-Like Receptor 4 (TLR4), IL-6 (Interleukin-6), interleukin-1β (IL-1β), tumor necrosis factor-α (TNF-α), Vascular Endothelial Growth Factor (VEGF), and Nuclear Factor Kappa B (NF-κB) human ELISA Kit were purchased from Thermo Fisher Scientific (Life Technologies, Carlsbad, CA, USA).

### 2.2. Plant Material and Extract Preparation

*Althaea officinalis* leaves were obtained from the Haraz Company, which is based in Cairo, Egypt. The leaves were dried in the shade, ground into a fine powder, and extracted after purchase. The choice of marshmallow leaves instead of roots was due to the presence of bioactive compounds such as flavonoids, phenolic acids, and mucilage, which are highly effective in treating oral ulcers. The leaves are rich in polysaccharides and other compounds that are beneficial for oral mucosal healing. To extract the leaves, a Soxhlet apparatus was used, with 300 milliliters of methanol–water mixture (8:2) as the extraction solvent. The total amount of powdered leaves used was 100 g. After twelve hours of extraction, the extract was transferred to glass containers. For 24 h, the extract was heated in an oven at 50 degrees Celsius. The final dried extract was stored at 4 degrees Celsius until ready for use [3]. Methanol was chosen over ethanol as the extraction solvent because it has better solvent properties that make it easier to extract both hydrophilic and hydrophobic compounds. This includes phenolic acids and flavonoids, which are known to have anti-inflammatory and antimicrobial properties.

### 2.3. Metabolic Profiling Using LC/MS/MS

#### 2.3.1. Sample Preparation

A 50 mg portion of the freeze-dried methanolic extract was dissolved in 1 mL of a solvent mixture composed of distilled water, methanol, and acetonitrile in a 2:1:1 ratio. To ensure complete dissolution, the solution was thoroughly vortexed before being ultrasonically sonicated at 30 kHz for 10 min. A 20 μL aliquot of the stock solution was diluted with 1 mL of the same solvent mixture and then centrifuged at 10,000× *g* rpm for 5 min to create the working solution. A 10 μL aliquot of the resulting 1 μg/mL sample was injected into the LC-MS/MS system, utilizing negative ions for analysis [28].

#### 2.3.2. LC/MS/MS Analysis

The LC-MS/MS analysis was conducted using a high-resolution quadrupole time-of-flight mass spectrometer (Q-TOF-MS) (Xevo^®^-G2-XS Q-TOF, Waters, Milford, MA, USA) equipped with a Duo-Spray ion source and operated in negative electrospray ionization mode. The analysis was conducted using a tandem mass spectrometer integrated with a high-performance liquid chromatography system that included an autosampler, an in-line filter disk pre-column (0.5 μm × 3.0 mm), and a C18 analytical column. Chromatographic separation was conducted at a flow rate of 300 μL/min and a column temperature of 40°C. The mobile phase comprised two solutions: Solution A consisted of 5 mM ammonium formate (NH_4_HCO_2_) with 1% methanol (MeOH), adjusted to pH 8 using sodium hydroxide, and Solution B comprised solely acetonitrile (ACN). A gradient elution program commenced with a 10% Solution B for the initial 20 min. Between 21 and 25 min, the proportion of Solution B increased consistently to 90% [29]. The composition was subsequently reduced to 10% Solution B between 25.01 and 28 min, followed by re-equilibration at 90% Solution B.

#### 2.3.3. Mass Spectrophotometry

Table 1 show the high-resolution quadrupole time-of-flight mass spectrometer with a Duo-Spray source and negative electrospray ionization (ESI) mode was used for the mass spectrometric analysis. The source temperature, declustering potential, and capillary voltage were all set to 600 °C, and −4500 V Gases 1 and 2 maintained constant pressures of 40 psi. Information-dependent acquisition (IDA) was used to obtain the MS and MS/MS spectra over a mass range of 50–1100 *m*/*z*. The collision energy was −35 V, with a spread of 20 V and an ion tolerance of 10 ppm. The survey scan time was 50 milliseconds, and the 15 most common ions were chosen for MS/MS fragmentation for each scan [30].

A comprehensive negative ion database containing over 1500 entries was utilized to process and analyze the data in conjunction with specialized metabolomics software. The analysis used a minimum peak height of 100 amplitude units, a mass slice width of 0.05 Da, and a smoothing level of two scans. Mass tolerances of 0.01 Da and 0.05 Da were chosen for MS1 and MS2, respectively. The identification parameters were established with a retention time tolerance of 0.05 min and an MS1 tolerance of 0.25 Da. The alignment parameters were established with a retention time tolerance of 0.05 min and an MS1 tolerance of 0.2 Da.

### 2.4. Experimental Design Construction of M-LNs

Preliminary research identified factors that may significantly affect the SLN characteristics. The 34-trial factorial design and version 13 of Design Expert^®^ software (Stat-Ease, Minneapolis, MN, USA) were used to optimize the formulation with M-SLNs. The investigation focused on four variables. The factors were lipid concentration (A), SAA concentration (B), lipid type (C), and SAA type (D). Table 2 lists the polydispersity index (Y2), zeta potential (MV) (Y3), encapsulation efficiency (%) (Y4), and particle size (nm) (Y1) as responses (dependent variables). A statistical study was conducted using analysis of variance (ANOVA) at a 95% level of significance (*p* < 0.05) to estimate the data received and the impact of the variables on M-SLN preparation.

### 2.5. Preparation of Marshmallow–Clove Oil Loaded Solid Lipid Nanoparticles

M-CO-SLNs were created through an enhanced method involving high-speed stirring and ultrasonication. Capric acid, stearic acid, and linoleic acid were regarded as solid lipids. Upon heating the lipid and drug mixture to 80 degrees Celsius, the oil phase was established. The mixture was transparent and uniform. Ten milliliters of deionized water and a weighed surfactant (poloxamer^®^ 403, poloxamer^®^ 188, or span^®^ 60) made up the aqueous phase [37]. At the same temperature, 80 °C heat was applied to the lipid and aqueous phases. The mixture was further stirred for ten minutes using a high-speed magnetic Heidolph stirrer (RZR 1) (Heidolph Instruments, Schwabach ,Germany) at a stirring speed of 1000 rpm to form a stable pre-emulsion after including the aqueous phase dropwise into the lipid phase. For the ultrasonic treatment of a pre-emulsion of clove oil (CO.) and water for five minutes with a probe (Sonics and Materials Inc., Sonics, India), the sonication process was carried out at a frequency of 20 kHz and 500 watts for 5 min at an intensity of 50% power, with a pulse cycle of 5 s on and 10 s off to ensure proper emulsification. At last, the hot dispersion was refrigerated at 4 °C and kept for further study [5].

### 2.6. Investigation of Marshmallow–Clove Oil Loaded Solid Lipid Nanoparticles

#### 2.6.1. Measurement of Particle Size, Polydispersity Index, and Zeta Potential

The M-CO-SLNs were analyzed using dynamic light scattering using a Malvern Nano-ZS device (Malvern Instruments Ltd., Malvern, UK) at 25 ± 2 °C to assess the vesicular size and polydispersity index. To minimize the multiple scattering effects, the samples were diluted in deionized water at a 1:10 (*v*/*v*) ratio prior to analysis. The zeta potential, indicative of vesicle surface charge and dispersion stability, was assessed using laser Doppler anemometry with the same equipment. Each experiment was conducted three times, and the mean values along with standard deviations (SDs) were documented [26,38].

#### 2.6.2. Percentage of Encapsulation Efficiency (EE%)

Unentrapped marshmallow was isolated from the solid lipid nanoparticle suspension using ultrafiltration in centrifuge tubes (Sartorius Vivaspin, MW cut-off 3000; Sartorius Stedim Biotech Company Ltd., Aubagne, France). Centrifugation was conducted at 20,000 rpm for 20 min at 4 °C to achieve the complete separation of the free drug [39,40]. The EE% was calculated using the following formula:

(1) $$\mathrm{EE}\%\frac{Amount\;of\;Mashmallow\;entrapped\;in\;SLNs}{Total\;Mashmallow}\times 100$$

#### 2.6.3. Optimization of Marshmallow–Clove Oil Loaded Solid Lipid Nanoparticles

The desirability function was employed to identify the optimal formula. The optimization parameters were set to select the lowest P.S and PD, as well as the maximum EE% and ZP (absolute values). The trial with the highest desired function under the given constraints was identified as the most suitable formula for further investigation.

### 2.7. Improvement of Optimized M-CO-SLNs

#### 2.7.1. Transmission Electron Microscopy (TEM) Analysis

To evaluate the morphological properties of the M-CO-SLN formulation, transmission electron microscopy (TEM) analysis was selected. The morphological structural properties of M-CO-SLNs were investigated using a JEM-1010 transmission electron microscope (JEOL, Tokyo, Japan). Before examination, the samples were positively stained with 1% (*v*/*v*) uranyl acetate. The microscope was operated at an acceleration voltage of 80 kV, with magnifications ranging from 10,000 to 100,000 times [41].

#### 2.7.2. Fourier Transform Infrared (FTIR) Spectroscopy Analysis

The analysis of pure marshmallow, clove oil, blank SLNs, and the optimal formula (M-CO-SLNs) was conducted utilizing FTIR (Jasco, FT/IR 6100, Tokyo, Japan). Each of these was assessed on an individual basis as well. Each sample consisted of two milligrams of anhydrous KBr. Subsequently, the amalgamation was subjected to compression into a disk and maintained at an ambient temperature and pressure, and the disk exhibited a range of 400 to 4000 cm^−1^ [42].

#### 2.7.3. Analysis of Differential Scanning Calorimeters (DSCs)

Using a DSC-60 (Shimadzu Corporation, Kyoto, Japan) with indium (m.p = 156.6 °C, purity = 99.99%) at a 10 °C/min heating rate, the DSC thermogram illustrates the thermal properties of pure marshmallow, clove oil, blank SLNs, and the optimal formula (M-CO-SLNs) formulations. This study offers insights into the crystalline and thermal stability of the formulations [37].

#### 2.7.4. In Vitro Release Study

An analysis was performed to determine the amount of M. that was released from the M. solution and compared to M-CO-SLNs. The formulations were placed in a dialysis bag with a cut-off of 12–14 kDa to be processed. In the following step, the bags were submerged in PBS (pH 7.4, 500 mL). At intervals that had been determined in advance, aliquots of the release medium containing 1 milliliter were extracted and replaced with fresh PBS of the same volume. According to what was discussed earlier in Section 2.3, the amount of M. that was released was measured using LC/MC for a period of up to 72 h. The experiment was carried out three times to ensure accuracy [21].

#### 2.7.5. Evaluation of Stability

Solid lipid nanoparticles were maintained at 4 ± 2 °C for six months to evaluate their long-term stability. The vesicle dimensions, polydispersity index (PDI), and zeta potential were measured at predetermined intervals (0, 3, and 6 months) using the techniques described in Section 2.3. Measurements were taken three times to ensure reliability, and the results were reported as mean ± standard deviation (SD) [2].

### 2.8. Preparation of M-CO-SLNs Sponges

To produce a collagen solution that has been subjected to heat denaturation, five grams of collagen powder were dissolved in five hundred milliliters of distilled water, while the pH of the solution was kept at that level. It is necessary to bring the solution up to a temperature of fifty degrees Celsius in ten minutes [43]. After that, one N sodium hydroxide was added to bring the pH level of the solution up to 7.0. After that, the refined M-CO-SLN dispersion was added to the collagen solution, and it was stirred continuously for four hours to guarantee that the mixture was homogenous. After the mixture was poured into a mold, it was frozen at 85 degrees Celsius for four hours, and then it was lyophilized for twenty-four hours. This was carried out to make the collagen sponge. A glutaraldehyde solution containing 0.25 percent by weight was used to cross-link collagen sponges, which was then incubated at 4 degrees Celsius for twenty-four hours. The preparation of control sponges that were devoid of M-CO-SLNs was carried out in the same manner as before for comparative analysis [44].

#### 2.8.1. Characterization of M-CO-SLNs Sponges

##### Morphological Characterization of M-CO-SLNs Sponges

A scanning electron microscope (JSM-6360, JEOL, Tokyo, Japan) helped to assess the shape of the M-CO-SLN sponge. Before the testing, the samples were gold-coated. About 1 mg of the sample was adhered to a receptacle using a double-sided adhesive strip. SEM images were acquired using a 15 kV accelerating voltage [12].

##### Fourier Transform Infrared (FTIR) Spectroscopy Analysis

The Fourier Transform Infrared Spectroscopy spectra for M-CO-SLN sponges and blank sponge using the same method are mentioned before in Section 2.7.2 [45].

##### Analysis of Differential Scanning Calorimeters (DSCs)

The differential scanning calorimetry thermograms for M-CO-SLN sponges and blank sponge using the same method are mentioned in Section 2.7.3.

##### Porosity Assessment

To determine the porosity of collagen and the M-CO-SLN sponges, each sample was submerged in an ethanol-filled measuring cylinder for a period of twenty-four hours. To determine the porosity, Equation (2) was utilized. In this equation, ρ represents the density of ethanol, Wf represents the final weight of the wetted sponge, and Wi represents the initial weight of the sponge [46].

(2) $$\mathrm{Porosity}=\frac{Wf-Wi}{\rho \left(ethanol\right)/V}\times 100$$

##### Swelling Ratio

Collagen and M-CO-SLN-loaded sponges were immersed in PBS (pH 7.4) and PBS (pH 5.5), respectively, and incubated at 37 °C. At predetermined intervals (1, 3, and 7 days), the sponges were removed, and the wet weight was determined by gently blotting off any excess buffer. The swelling ratio was calculated using Equation (3), where Wi is the sponge’s initial weight and Ww is the wet weight after removal from either PBS (pH 7.4) or PBS (pH 5.5) [47].

(3) $$\mathrm{Swelling}\;\mathrm{ratio}=\frac{Ww-Wi}{Wi}$$

##### Mechanical Characterization

The Universal Testing Machine (WDW-20E, Jinan Time Shijin Group, Jinan, China) was used to investigate the mechanical properties of collagen sponges while it was both unloaded and loaded with M-CO-SLNs. This was carried out to determine the sponges’ mechanical properties. The tensile strength and elongation percentage were two of the mechanical properties that were investigated in this study [10]. At room temperature, samples with dimensions of 100 mm by 20 mm were pulled at a crosshead speed of forty millimeters per minute until they ruptured. Equations (4) and (5) were utilized to ascertain the tensile strength of the material as well as the percentage of elongation that occurred after the break in the material [48].

(4) $$\mathrm{Tensile}\;\mathrm{strength}=\frac{Load}{Sample\;length*width}\;$$

(5) $$\mathrm{Elongation}=\;\frac{Displacment\;at\;break}{Sample\;length}\times 100\;$$

### 2.9. Antimicrobial Study

#### 2.9.1. Diffusion Agar Method

The antimicrobial efficacy of M-CO and M-CO-SLNs was initially assessed using standardized bacterial strains supplied by the Al-Azhar University Regional Center for Mycology and Biotechnology. A suspension with a calibrated concentration of 0.5 McFarland (1.5 × 10^8^ CFU/mL) was generated by incubating the microbial strains *Escherichia coli* ATCC 25922, *Pseudomonas aeruginosa* ATCC 27853, and *Candida albicans* RCMB 005003 (1) ATCC 10231 at 37 °C for 18 to 24 h, and the solutions were subsequently incorporated into agar. Separate aliquots were carefully extracted from ten microliters of various diluted formulations. The agar plates were incubated at 37 °C for 24 h, following the established droplet adsorption protocol. The presence of an inhibitory zone around the application site suggests antibacterial efficacy. The experiment was repeated three times, measuring and documenting the diameter of the inhibitory zone and analyzing the selected results to determine their clarity [21].

#### 2.9.2. Assessment of the Minimum Inhibitory Concentration (MIC)

The minimum inhibitory concentration (MIC) is the smallest concentration required to inhibit detectable microbial growth. A lower MIC value indicates that the corresponding agent has better antimicrobial activity. As a result, the MIC is an effective metric for comparing the efficacy of various antimicrobial agents or microbial strains. During the following experimental phase, the MIC was determined using the microdilution technique. Isolated microorganisms were grown on Mueller–Hinton Agar (MHA) at 37 °C until they entered the exponential growth phase. To dilute the formulations, 100 µL of the bacterial strains from the Regional Center for Mycology and Biotechnology (RCMB) were added to each well of the flat-bottomed, 96-well microplates, except for the first row, which had 200 µL of the oils. The results were shown as the mean ± SD [3].

### 2.10. In Vivo Study

#### 2.10.1. Induction of Type 1 Diabetic Rat Model

**Figure 1:**
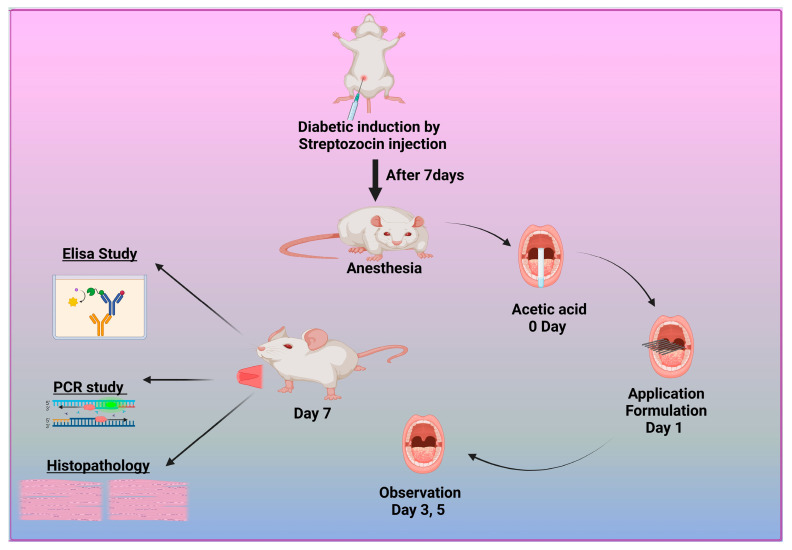
Diagram illustrating the methodology for inducing and treating diabetic oral ulcers.

Adult male albino Wistar rats were used to assess the efficacy of the prepared formulations. Forty mature male albino rats weighing between 180 and 200 g and aged 8 to 12 weeks were obtained from the Nahda University animal house, which is located at the Faculty of Pharmacy in Nahda, Egypt. The subjects acclimated to the standard environmental conditions for one week, encompassing a natural light/dark cycle, a constant temperature of 22–25 °C, and a relative humidity of 45–60%. During the study, the rats had unrestricted access to water and were provided with regular pellet meals in plastic wire mesh cages. All protocols and animal care procedures were carried out under the Animal Ethics Committee guidelines of Nahda University Faculty of Pharmacy in Beni-Suef, Egypt (Approval NUB-025-041). Ketamine was used to induce anesthesia in the animals at a dose of 80 mg/kg. The rats were given 1% STZ intraperitoneally after a 12 h fast. Their blood glucose levels were randomly measured one week after injection using a rat tail vein sample. Rats with blood glucose levels greater than 16.7 mM were identified as effective models for type 1 diabetes [2]. This threshold indicated the successful simulation of the disease, as shown in Figure 1.

#### 2.10.2. Induction of Diabetic Oral Ulcers

To create the oral mucosa ulcer model in rats, we used a modified traditional approach. After anesthetizing the diabetic albino Wistar rats, the mandibular mucosa was dried with sterile cotton. We soaked square-shaped filter paper (4 mm × 4 mm) in 50% acetic acid for 6 s. It was then placed directly on the mucosa [49]. After 40 s, the filter tissue was removed, and the constructed area was cleaned with a sterile cotton ball soaked in PBS to remove any remaining acetic acid. The resulting ulcers were divided into five groups: one baseline cohort received PBS, the second was left untreated, and the remaining three were treated with M-CO, M-CO-SLNs, and M-CO-SLN sponges, respectively. After a 7-day medication regimen, oral ulcer symptoms were visually observed and recorded, including the number and duration of ulcers, and the size of the ulcers in each group was measured with vernier calipers, as shown in Figure 1.

#### 2.10.3. Enzyme-Linked Immunosorbent Assay (ELISA)

All animals were anesthetized with pentobarbital sodium (200 mg/kg, IP) 48 h after the previous treatment to collect blood samples through the retroorbital sinus and scarify them via cervical dislocation. The blood samples were centrifuged and serum tested for TLR4, IL-6, IL-1β, TNF-α, VEGF, and NF-κB (Life Technologies, CA, USA). All things considered, it is a plate-based testing technique that is meant especially to measure and identify molecules, including proteins, peptides, hormones, and antibodies [50]. The most often used ELISA system is the sandwich test. The name “sandwich” test comes from the analyte under investigation in this capture assay being sandwiched between the capture and detection main antibodies. The adaptability and durability of sandwich construction make it appealing. The optical density and observed biomarker levels have a direct correlation, as shown by this method.

#### 2.10.4. RNA Extraction and Real-Time PCR Method

##### RNA Extraction and Reverse Transcription

The RNeasy Mini Kit (Catalog no. 74104), QIAGEN, Hilden, Germany. extracts the total RNA per the manufacturer’s instructions. Extraction used deionized water (DDW) and Sigma-Aldrich ß-Mercaptoethanol, diluted to 70% with ethanol (96%, Applichem, Darmstadt, Germany). Using a TissueLyser (Qiagen), RNA was extracted from 30 mg of tissue samples homogenized in Buffer RLT with 10 µL/mL of ß-Mercaptoethanol. The samples were then centrifuged and purified with RNeasy spin columns. Following RNA elution in RNase-free water, on-column DNase digestion effectively eliminated any residual DNA. Reverse transcription of 1 μg of total RNA was performed in two steps, utilizing Revert Aid Reverse Transcriptase (Thermo Fisher Scientific, Waltham, MA, USA (Cat. No. EP0441)) along with random hexamer primers. We synthesized additional complementary DNA (cDNA) for quantitative real-time polymerase chain reaction (qRT-PCR) analysis [37].

##### The qRT-PCR Gene Expression Assay

Table 3 displays the sequences of the Quantitect SYBR Green PCR Kit (Cat. No. 204141, Qiagen) and specific primers used to quantify IFNG (Interferon Gamma) and Insulin-like growth factors’ (IGF-1) gene expression. In each reaction, 2.5 µL of 2 × Quantitect SYBR Green PCR Master Mix, 50 picograms of forward and reverse primers, 0.25 µL of reverse transcriptase, 8.25 µL of RNase-free water, and 3 µL of complementary DNA were used. A Stratagene MX3005P real-time PCR study examined the IFNG and IGF1. The cycling trial began with 30 min of 50 °C reverse transcription and 15 min of 94 °C DNA denaturement. Each of the 40 amplification cycles had 15 s of denaturation at 94 °C, 30 s of annealing at 60 °C, and 30 s of extension at 72 °C, and the procedure ended with a 5 min 72 °C extension. Gene expression was standardized using the 2^−ΔΔCt^ method, using Ubiquitin 5 as the reference gene. The Stratagene MX3005P software analyzed duplicate sample Ct values. The amplification curves and threshold cycle values (Ct) were compared between the experimental and control groups to assess the gene expression changes [51].

### 2.11. Histopathological Examination

Following the administration of various formulations for seven days, the mucosal tissues of rats belonging to each group were removed, fixed with 4% paraformaldehyde for forty-eight hours, dehydrated with ethanol, transparentized with xylene, embedded in paraffin, and then cut into slices. To observe the changes in the pathological morphology, H&E staining was utilized, and the tissue was then deparaffinized for standard examination using a light electric microscope with a light microscope [54].

### 2.12. Statical Analysis

The stability and comparison studies were statistically analyzed using one-way ANOVA, Tukey–Kramer multiple-comparison tests, and Student’s t-tests among different groups. GraphPad Prism version 8 was used to compare the groups using the Student’s t-test (*p* < 0.05). The results were presented as the means ± SD.

## 3. Results and Discussion

### 3.1. LC/MS/MS Analysis of Marshmallow (M.) Leaf Extract

**Figure 2:**
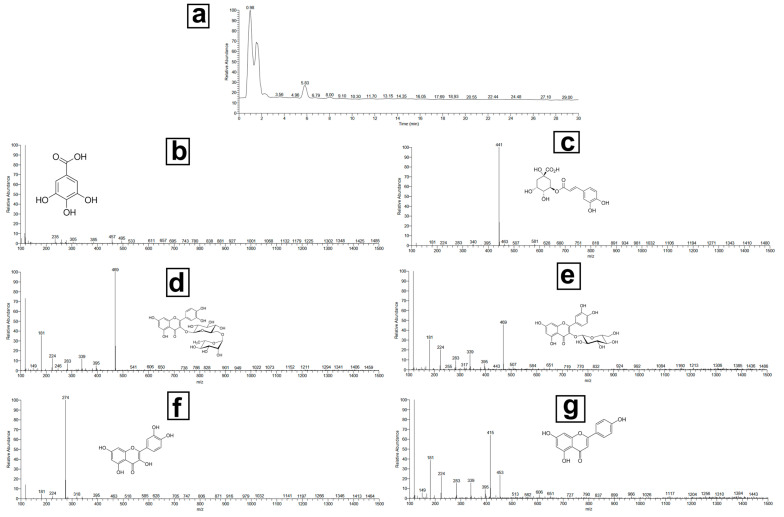
Total LC/MS/MS analysis of the methanolic extract of marshmallow leaves: (**a**) full chromatogram, (**b**) gallic acid, (**c**) chlorogenic acid, (**d**) rutin, (**e**) isoquercitrin, (**f**) quercetin, and (**g**) apigenin.

LC-MS/MS analysis revealed several key bioactive compounds in the marshmallow extract, including gallic acid, chlorogenic acid, rutin, isoquercitrin, quercetin, and apigenin metabolites, known for their antimicrobial, anti-inflammatory, and wound-healing properties. While quantitative analysis was not the focus of this study, the identification and profiling of these metabolites provide critical insights into the therapeutic potential of marshmallow. Through ultra-performance liquid chromatography coupled with mass spectrometry (UPLC-MS/MS), we offered a comprehensive metabolic fingerprint of marshmallow leaves, laying the groundwork for future quantitative and functional studies. The M. leaf phenolic acids and flavonoids in the methanolic extract showed anti-inflammatory and antibacterial properties. All the identified metabolites are displayed in Table 1, along with the retention durations, peak positions of molecular ions, and primary fragment ions. Figure 2 shows the typical MS/MS spectra of the chosen compounds. The antioxidant properties of phenolic acids make them an important part of the pharmaceutical activity of medicinal plants. This was the first time that gallic acid and chlorogenic acid were found in M. Gallic acid (*m*/*z* 169.01) exhibited fragmentation patterns at *m*/*z* 125 and *m*/*z* 79, indicating the loss of CO_2_ and further breakdown of the core structure. At *m*/*z* 191, 179, and 173, chlorogenic acid (*m*/*z* 353.09) generated unique fragment ions. This is in line with earlier results on the fragmentation mechanisms of hydroxycinnamic acid derivatives. M. is high in flavonoids, bioactive molecules with anti-inflammatory and antioxidant action. The methanolic extract revealed rutin, isoquercitrin, quercetin, and apigenin, according to the study. Confirming its glycosylated structure, rutin (*m*/*z* 609.14) displayed fragmentation at *m*/*z* 301 (quercetin core) and *m*/*z* 179. Fragments at *m*/*z* 301 (quercetin), *m*/*z* 179, and *m*/*z* 151 in isoquercitrin (*m*/*z* 463.09) suggested that the glucoside had cleaved. At *m*/*z* 179 and 151, the compound quercetin (*m*/*z* 301.03) produced notable fragment ions, with pieces at *m*/*z* 117 and 151 that matched its known flavone structure, apigenin (*m*/*z* 269.04). The chemical makeup of marshmallow (M.) leaves is examined in this work to identify the phenolic acids and flavonoids possibly beneficial for human health. The chemicals in the plant help to support its conventional medical applications. Among these are gallic acid, chlorogenic acid, rutin, isoquercitrin, quercetin, and apigenin [55]. Although marshmallows are rich in mucilage, which contributes significantly to mucoprotective properties, the current study was specifically designed to identify and characterize the phenolic and flavonoid compounds associated with antimicrobial and wound-healing effects. Mucilage analysis requires distinct extraction and analytical methods (e.g., gravimetric quantification), which were beyond the present scope. Future investigations will aim to quantify and evaluate the mucilage content to fully exploit its therapeutic potential.

### 3.2. Optimization of Factorial Design

The factorial design was employed to optimize the formulation of M-CO-SLNs, considering four independent variables: A: lipid concentration, B: surfactant concentration, C: lipid type, and D: surfactant type. The findings indicated that the dependent responses were markedly affected by the variables of Y1: particle size (P.S), Y2: polydispersity index (PDI), Y3: zeta potential (ZP), and Y4: entrapment efficiency (EE%), as shown in Table 2. The model demonstrates its reliability through impressive R^2^ values ranging from 0.9775 to 0.9886, which signifies a robust correlation between the predicted and observed values. The data presented in Table 4 and Table 5 reveal that the adjusted and predicted R^2^ values vary by less than 0.2, suggesting that the model demonstrates a high level of accuracy and robustness. The precision values were higher than the acceptable level of four, which means that the model is ready to be optimized.

#### 3.2.1. The Influence of Formulation Parameters on Particle Size (P.S)

**Figure 3:**
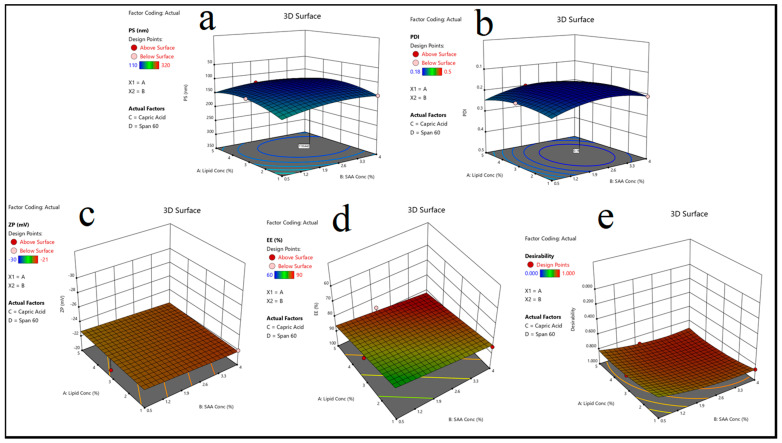
(A) 3D surface plot showing the effect of lipid concentration (Lipid Conc), (B) surfactant concentration (SAA Conc), (C) lipid type, and (D) surfactant type on the following responses: (**a**) particle size (P.S), (**b**) polydispersity index (PDI), (**c**) zeta potential (ZP), (**d**) entrapment efficiency (EE%), and (**e**) desirability.

The particle size significantly influences the stability and bioavailability of solid lipid nanoparticles (SLNs). The results indicated that a rise in lipid concentration (Factor A) led to an increase in particle sizes, as demonstrated in Table 2. This trend is due to the higher viscosity of the dispersion medium, which reduces the efficiency of homogenization, resulting in larger particle aggregates. Furthermore, the type of lipid used had a significant effect on the PS, with formulations containing stearic acid exhibiting the largest particle size, followed by linoleic acid and capric acid. This observation can be attributed to stearic acid’s higher melting point and hydrophobic nature, which promote aggregation. Furthermore, the type and concentration of surfactants (SAA) influenced the PS, with poloxamer 407 producing smaller particles than span 60 [56], most likely due to its superior steric stabilization properties, as shown in Figure 3a.

#### 3.2.2. Impact of Formulation Variables on Polydispersity Index (PDI)

The PDI measures the particle size distribution uniformity; lower values indicate a more homogeneous system. The findings revealed that increasing the SAA concentration (Factor B) resulted in a lower PDI, implying that the nanoparticle system is more stable, as shown in Table 2. The formulations containing poloxamer 407 had the lowest PDI values, indicating that they were effective at preventing particle aggregation [57]. In contrast, formulations containing span 60 had slightly higher PDI values, which could be attributed to its limited solubility and emulsifying capacity, as shown in Figure 3b.

#### 3.2.3. The Effect of Formulation Variables on Zeta Potential (ZP)

The zeta potential is a critical parameter for determining nanoparticle stability, with higher absolute values indicating greater electrostatic repulsion and stability, as shown in Table 2. The findings revealed that the lipid type had a significant impact on ZP, with stearic acid-based formulations having the highest negative ZP values. This finding is consistent with stearic acid molecules’ strong electrostatic charge, which contributes to increased repulsion between nanoparticles. Furthermore, formulations containing poloxamer 188 and span 60 had slightly lower ZP values than those containing poloxamer 407, indicating differences in the surface-active properties [58], as illustrated in Figure 3c.

#### 3.2.4. The Impact of Formulation Variables on Entrapment Efficiency (EE%)

Entrapment efficiency is a critical factor in drug loading and release behavior. The findings revealed that increasing the lipid concentration (Factor A) increased the EE% by creating a larger matrix for drug entrapment, as shown in Table 2. Linoleic acid-based formulations had the highest EE% of all lipid types, possibly due to their unsaturated structure, which allows for better drug molecule accommodation. Surfactant type also played an important role, with poloxamer 407-based formulations achieving the highest EE%, most likely due to its stabilizing effect on the lipid core, which prevents drug leakage, as shown in Figure 3d. The HLB value is essential for emulsification and nanoparticle stability as it influences the self-assembly and dispersion of lipid-based systems. Based on the formulation composition [5], HLB values were found for several different combinations of lipids and surfactants. Because capric acid does not repel water very well, stable formulations that contain it need poloxamer 407 and other surfactants with high HLB values. Surfactants with lower HLB values, such as span 60, performed better in stearic acid formulations that were highly hydrophobic. The results revealed that formulations with the appropriate amounts of lipids and surfactants to achieve a balanced HLB value were more stable and uniform, as evidenced by a lower PDI and higher EE% [59].

#### 3.2.5. Identifying the Optimized M-CO-SLNs

The optimal M-CO-SLNs formulation (Run 16: 5% lipid concentration, 4% SAA concentration, capric Acid as lipid, and poloxamer 188 as SAA) was determined by the best combination of the PS, PDI, ZP, and EE%. The experimental values of the optimized formulation (P.S = 110 nm, PDI = 0.18, ZP = −24 mV, and EE% = 90%) were in close agreement with the predicted values, thereby validating the model’s reliability, as shown in Table 4. This formulation demonstrated optimal characteristics, including small particle size, uniform distribution, high stability, and superior drug entrapment efficiency, positioning it as the most suitable candidate for future research, as shown in Figure 3e.

### 3.3. Improvement of Optimized M-CO-SLNs

#### 3.3.1. Transmission Electron Microscopy (TEM) Analysis

**Figure 4:**
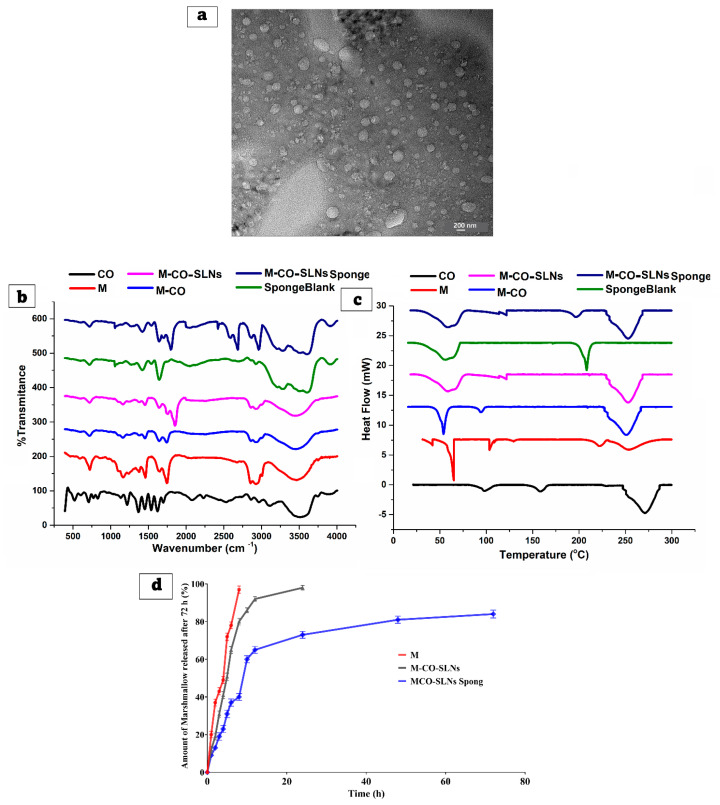
Scanning electron microscope (SEM) (**a**), Fourier Transform Infrared (**b**), analysis of differential scanning calorimeters (**c**), and in vitro release (**d**) from various developed marshmallow (M.) solutions, marshmallow–clove oil solid lipid nanoparticles (M-CO-SLNs), and marshmallow–clove oil solid lipid nanoparticle (M-CO-SLN) sponges.

The transmission electron microscopy (TEM) examination was used to establish the morphological examination of M-CO-SLNs, as indicated in Figure 4a. The size of the nanoparticles was consistent throughout, and they had a spherical shape, as they did not accumulate. Therefore, the average P.S that was obtained by the zeta sizer had a perfect correlation with the findings that were obtained from the TEM.

#### 3.3.2. Fourier Transform Infrared (FTIR) Spectroscopy Analysis

Figure 4b shows the chemical interactions and structural changes in marshmallow (M.), clove oil (CO), and marshmallow–clove oil solid lipid nanoparticles (M-CO-SLNs) that were investigated using FTIR. The obtained spectra clarify the existing functional groups and possible molecular interactions among the components. The FTIR spectrum of M. revealed clear peaks that corresponded to its main functional groups. The broad peak around 3200–3600 cm^−1^ is caused by O-H stretching vibrations, which indicate the presence of hydroxyl groups in polysaccharides, flavonoids, and other bioactive compounds. The peaks observed at 1600–1700 cm^−1^ signify C=O stretching linked to carboxyl and ester groups [60]. The fingerprint region below 1500 cm^−1^ features numerous bands that illustrate the intricate structure of marshmallow, including C-O and C-H bending vibrations. CO exhibited clear peaks for its primary components, especially eugenol. While eugenol shows a strong peak in the range of 3000–3100 cm^−1^, ester groups show C=O stretching. Based on the C-O stretching band seen at 1200–1300 cm^−1^, phenolic groups appear to be present. The FTIR spectrum allows one to evaluate the interactions of CO with other formulation components [12,61]. The FTIR spectrum of M-CO-SLNs showed significant shifts and intensity changes when compared to the individual components, indicating that M and CO were successfully encapsulated within solid lipid nanoparticles. Broadening and somewhat shifting the characteristic O-H stretching band revealed that hydrogen bonds interact between the lipid matrix and bioactive components. Confirming their inclusion in the lipid system, the lower intensity of the C=O and C-O peaks from M. and CO. suggests a reduction in their free-state availability [62]. The FTIR analysis verifies the successful encapsulation of M. and CO., which are subsequently integrated into the M-CO-SLNs, indicating substantial molecular interactions, enhanced stability, and prospective applications in controlled release.

#### 3.3.3. Analysis of Differential Scanning Calorimeters (DSCs)

Figure 4c shows that the DSC was used to assess the thermal properties of marshmallow (M.), clove oil (CO.), and marshmallow–clove oil solid lipid nanoparticles (M-CO-SLNs). The obtained thermograms reveal information about phase transitions, crystallinity, and potential interactions between the components. The DSC thermogram of pure M. displays several thermal events, indicated by the multiple endothermic peaks observed. The degradation of bioactive compounds and polysaccharides at elevated temperatures may lead to further thermal transitions within the marshmallow matrix. The existence of bound water likely accounts for the detected endothermic peak between 50 and 100 °C. The intricate thermal behavior of marshmallow reveals that it consists of various components, each playing a role in the observed transitions. Clove oil (CO.) displayed an endothermic peak in the 200–250 °C range, suggesting the evaporation or degradation of its volatile components, especially eugenol. Clove oil can exist in an amorphous form free from abrupt melting transitions. The factor most affecting the stability of clove oil and its possible interactions with other formulation components is the thermal profile. The thermogram of M-CO-SLNs showed a wide endothermic peak within the temperature range of 50 to 100 °C, ascribed to the melting of the lipid matrix used in the formulation of the nanoparticles. Variations in thermal behavior relative to pure M. and CO. point to the effective encapsulation of lipid nanoparticles. The reduced intensity of the characteristic peaks for CO. and M. suggests molecular interactions between the lipid matrix and the bioactive compounds, thus promoting increased stability and lower crystallinity. The DSC findings demonstrate that M. and CO. were successfully generated and incorporated into the M-CO-SLNs. The enhanced thermal characteristics suggest that sponges infused with M-CO-SLNs may function as a reliable and thermally stable drug delivery system, potentially enhancing their stability and bioavailability [63].

#### 3.3.4. In Vitro Release Study of Solid Lipid Nanoparticles

Figure 4d shows that the release profiles of (M.) solutions and (M-CO-SLNs) formulations were examined over 72 h to determine their sustained release properties. The drug release from the (M.) solution was rapid, reaching approximately 97% within 8 h, indicating that the drug was readily available in the solution and released immediately. This indicates that M. solutions lack a sustained-release mechanism, making them unsuitable for long-term drug delivery applications. The M-CO-SLN formulation had a more controlled and sustained release profile. Initially, 12% of the drug was released within one hour, which increased to 50.76% after five hours and 92% after twelve hours. In comparison to the M. solution, achieving 98% within 24 h suggests a slower and longer drug release profile. This suggests that enclosing the drug within SLNs effectively reduced the initial burst effect, allowing for a gradual release of the drug over time. M. solutions dissolve fast, since no lipid barrier exists, and M-CO-SLNs show a controlled release of drugs resulting from their encapsulation within lipid nanoparticles, thus preventing diffusion and extending the availability of the drug. The results show that M-CO-SLN formulations clearly extend drug release in comparison to M. solutions, thus reducing the need for regular administration and perhaps improving therapeutic efficacy [64].

#### 3.3.5. Evaluation of Stability

The six-month stability study of optimized marshmallow–clove oil solid lipid nanoparticles (M-CO-SLNs 16) examined the particle size (P.S), polydispersity index (PDI), zeta potential (ZP), and entrapment efficiency (EE%), as shown in Table 6. The nanoparticles’ physicochemical properties were significantly affected by the storage conditions, as the 4 °C samples were more stable than the 25 °C samples. Freshly prepared M-CO-SLNs 16 had a particle size of 110 ± 0.76 nm, PDI of 0.18 ± 0.05, zeta potential of −24 ± 0.32 mV, and entrapment efficiency of 90 ± 0.65%. A stable, well-dispersed nanoparticle system with high drug loading efficiency is shown. In three months of storage at 4 °C, the particle size decreased to 107 ± 0.32 nm, while the PDI and zeta potential remained stable at 0.18 ± 0.63 and −24 ± 0.65 mV, respectively. Lower temperatures did not disrupt the structural integrity or drug retention capacity of SLNs, as their entrapment efficiency was only slightly reduced to 89 ± 0.39%. Samples at 25 °C for three months showed a slight increase in particle size (108 ± 0.76 nm) and PDI (0.19 ± 0.24), indicating aggregation. The zeta potential decreased to −23 ± 0.11 mV, indicating a slight decrease in electrostatic stability. The drug entrapment efficiency dropped to 88 ± 0.19%, indicating leakage or degradation at higher storage temperatures. Variations persisted after six months, especially at 25 °C. Nanoparticle collapse or restructuring may have reduced the particle size to 104 ± 0.76 nm at 25 °C. Increasing the PDI to 0.20 ± 0.09 may indicate greater particle distribution heterogeneity. A decrease in the zeta potential to −21 ± 0.56 mV may lead to reduced particle repulsion and increased aggregation. Drug degradation or leakage may result from prolonged exposure to high temperatures, as the entrapment efficiency decreased to 85 ± 0.75%. After six months at 4 °C, M-CO-SLNs 16 demonstrated stability, with a particle size of 103 ± 0.76 nm and a PDI of 0.21 ± 0.87, indicating a slight increase. The zeta potential was measured at −21 ± 0.87 mV, reflecting a reduction comparable to that observed at 25 °C. The measured entrapment efficiency of 86 ± 0.98% indicates improved drug retention at lower temperatures. Over six months, M-CO-SLNs 16 are more stable at 4 °C than 25 °C. High-temperature storage may damage the nanoparticles, affecting formulation optimization in future studies. Changes in the minimal particle size, polydispersity index (PDI), and zeta potential indicate that M-CO-SLNs 16 have a good refrigeration shelf life for pharmaceutical applications [65].

### 3.4. Characterization of M-CO-SLN Sponge

#### 3.4.1. Morphological Characterization of M-CO-SLNs Sponge

**Figure 5:**
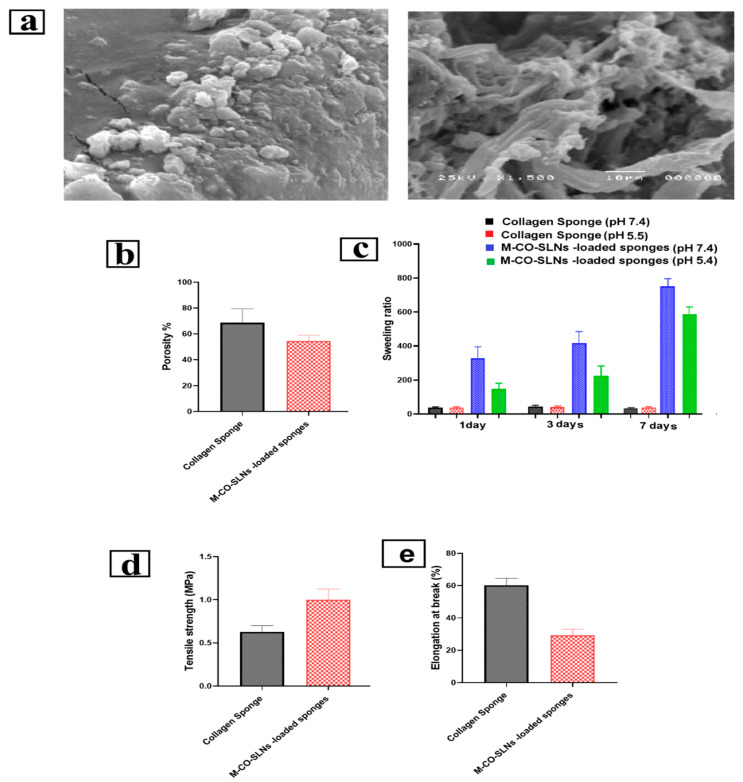
(**a**) Scanning electron micrograph (SEM) of M-CO-SLNs-loaded collagen sponge, (**b**) porosity of the M-CO-SLNs sponge assessed using the ethanol displacement technique, (**c**) swelling ratio quantified by soaking the sponge (with and without M-CO-SLNs) in PBS at pH 7.4 and pH 5.5 for time intervals of 1, 3, and 7 days, (**d**) mechanical properties of collagen sponge with and without M-CO-SLNs assessed in terms of tensile strength, and (**e**) percentage of elongation of collagen sponge samples.

The developed sponge’s porous structure is critical for creating an environment conducive to cell growth, proliferation, and migration. The morphology of the prepared M-CO-SLNs-loaded sponge was investigated using SEM imaging. Figure 5a depicts the prepared M-CO-SLNs-loaded sponge, which consisted of interconnected pore-forming thick sponges with honeycomb-like matrices. Because natural collagen degrades more rapidly than the cross-linked form, it was necessary to cross-link the sponge matrices with glutaraldehyde to preserve the integrity of the sponge structure [66]. In addition, it was discovered that the porous form of the sponge that was developed was suitable for imitating the extracellular matrix, which is beneficial for the regeneration of cells and the healing of infections of the mouth.

#### 3.4.2. Fourier Transform Infrared (FTIR) Spectroscopy Analysis

Figure 4b shows that the chemical interactions and structural changes in the blank sponge and M-CO-SLNs-loaded sponge were investigated using FTIR. The obtained spectra clarify the existing functional groups and possible molecular interactions among the components. The blank sponge collagen with glutaraldehyde showed clear peaks linked to the structural framework of collagen. Whilst the amide I band at 1650 cm^−1^ marks the C=O stretching of the peptide backbone, the amide II band at 1550 cm^−1^ corresponds with N-H bending and C-N stretching vibrations. These peaks confirm the structural integrity of collagen. The peak at 3300 cm^−1^ marks the proteinaceous character of collagen by the corresponding N-H stretching. With some peak shifts and intensity changes, the M-CO-SLNs-loaded sponge revealed a spectral profile matching M-CO-SLNs and the blank sponge. Strong hydrogen bonds forming between the collagen matrix and lipid nanoparticles are indicated by the widening point of O-H and N-H stretching [67]. The diminished intensity of the specific peaks associated with free marshmallow and CO signifies their effective absorption into the sponge. The observed spectral changes show that M-CO-SLNs were successfully integrated into the collagen sponge while maintaining structural integrity. The FTIR analysis verifies the successful encapsulation of M. and CO. within M-CO-SLNs before their incorporation into the collagen sponge. The observed spectral shifts and intensity variations suggest notable molecular interactions, increased stability, and possible applications in controlled release. The results validate the effectiveness of M-CO-SLN-loaded sponges as a delivery system for bioactive compounds [21].

#### 3.4.3. Analysis of Differential Scanning Calorimeters (DSCs)

Figure 4c shows that the DSC was used to assess the thermal properties of the blank sponge and M-CO-SLNs-loaded sponge. The obtained thermograms reveal information about phase transitions, crystallinity, and potential interactions between the components. The collagen cross-linked sponge demonstrated a notable thermal transition between 100 and 150 °C, presumably due to collagen denaturation. The peak indicates the thermal stability of the cross-linked collagen network and highlights the influence of glutaraldehyde on the structural properties of the sponge. The peak’s distinct characteristics indicate a semi-crystalline structure that maintains stability under thermal stress. The incorporation of M-CO-SLNs into the collagen sponge resulted in a DSC profile that shared characteristics with both the unmodified sponge and the M-CO-SLNs, albeit with significant shifts in the peak positions [68]. The minor decrease in the endothermic peak intensity points to interactions between nanoparticles and the collagen matrix controlling the general thermal behavior. Most likely, the interactions preserved the structural integrity of the sponge and allowed the encapsulated molecules to be released under control. The DSC findings demonstrate that M-CO-SLNs were successfully generated and incorporated into the collagen sponge. The observed thermal alterations and peak intensities suggest substantial interactions between the lipid nanoparticles and the sponge matrix, potentially enhancing the stability and prolonged release capabilities. The enhanced thermal characteristics suggest that sponges infused with M-CO-SLNs may function as a reliable and thermally stable drug delivery system.

#### 3.4.4. In Vitro Release Study of Sponges

The release profiles of M-CO-SLNs sponge formulations were examined over 72 h to determine their sustained release properties, as shown in Figure 4d. The M-CO-SLNs sponge formulation improved the overall release profile. Compared to M-CO-SLNs, the sponge formulation demonstrated an even longer drug release pattern, reaching only 9% at 1 h, 31% at 5 h, and 73% at 24 h, with 84% drug release achieved at 72 h. This suggests that the sponge formulation has a more enduring effect, making it a promising method of extended drug delivery. The drug’s release is further delayed by the porous structure of the M-CO-SLNs sponge, which serves as a reservoir. The results confirm that M-CO-SLNs sponge formulations significantly prolong drug release in comparison to M. solution, thereby reducing the necessity for frequent administration and potentially enhancing therapeutic efficacy. The sponge formulation has the longest release profile, making it ideal for long-term drug delivery applications [21].

#### 3.4.5. Porosity Assessment

Figure 5b shows that the collagen sponge had a porosity range of 58–87%, with an average porosity of 75–80%. The increased porosity primarily arises from the lyophilization process, which establishes an interconnected porous network that enhances fluid absorption and gas exchange, both of which are crucial for wound healing. The sponge’s porosity reduced slightly to a range of 49–60% after being loaded with M-CO-SLNs, with an average reduction of 65–70%. This decrease is anticipated because the nanoparticles partially occupy the sponge interspaces, which leads to a denser structure. Although the porosity decreased, it remained sufficient to facilitate the effective absorption of exudate and the permeability of oxygen, thereby creating a balanced moisture environment that facilitated the healing of mouth ulcers [69].

#### 3.4.6. Swelling Ratio

The capacity to swell is an essential characteristic of oral ulcer dressings, influencing their ability to maintain moisture, absorb exudates, and foster an ideal healing environment. An increased swelling ratio enhances fluid absorption and retention, which is essential for moist wound healing, as it facilitates epithelial cell migration and tissue regeneration. The collagen sponges that were prepared showed significant swelling ratios, which can be attributed to their cross-linked porous structure and hydrophilic characteristics. The significant water retention capacity indicates that these sponges can efficiently function as moisture-retentive wound dressings, helping to prevent wound dehydration and promote re-epithelialization. The swelling ratio increased over time, peaking by day 3, after which equilibrium was achieved. The free collagen sponge showed a greater swelling ratio than the M-CO-SLNs-loaded sponge. The decrease in the swelling capacity can be attributed to the presence of solid lipid nanoparticles (SLNs) within the collagen matrix, which partially fill the porous structure, thereby restricting water absorption. Despite this decrease, M-CO-SLNs-loaded sponges retained a high swelling capacity, absorbing exudate and hydrating the wound. Sponge swelling was strongly affected by the pH (Figure 5c). Collagen and M-CO-SLN sponges had higher swelling ratios at pH 5.5 than pH 7.4. For example, the collagen sponge had 281.66% swelling on day one and 726.66% by day seven. At pH 7.4, the swelling ratios dropped from 17.71% on day 1 to 20.88% on day 7. This suggests that collagen molecules protonate in an acidic environment (pH 5.5), causing electrostatic repulsion and matrix expansion, which increases water uptake [70]. At pH 7.4, decreased ionization near collagen’s isoelectric point reduces swelling, resulting in a smaller water absorption capacity [71]. The findings highlight the importance of optimizing the pH conditions to improve the swelling behavior, which is critical for wound healing applications involving moisture retention and exudate absorption. The experimental results support the observation that M-CO-SLNs-loaded sponges, while slightly less absorbent than pure collagen sponges, still had an adequate swelling capacity for ulcer management. Additionally, the increased swelling noted in acidic conditions indicates that these sponges could function best in wound environments, where the pH frequently leans toward being acidic because of inflammatory processes [72].

#### 3.4.7. Mechanical Characterization

The mechanical properties of mouth ulcer dressings play a vital role in their effectiveness, especially when it comes to treating oral ulcers. Flexibility, durability, and resistance to deformation are crucial elements in promoting patient comfort and facilitating effective healing. This study revealed notable variations in tensile strength and elongation at breaks between the pure collagen sponge and the M-CO-SLNs-loaded sponge, emphasizing the influence of solid lipid nanoparticles (SLNs) on the mechanical properties of the scaffold [73,74]. The tensile strength of the M-CO-SLN-loaded sponge was significantly higher than that of a pure collagen sponge. The collagen sponge’s tensile strength ranged from 0.54 to 0.75 MPa, while that of the M-CO-SLNs-loaded sponge ranged from 0.81 to 1.11 MPa. The increased mechanical strength is beneficial for oral ulcer applications because it provides better structural integrity and longer retention at the ulcer site. The strengthening properties of SLNs help the collagen matrix to stick together, making the dressing more resistant to biting, tongue movement, and saliva flow. M-CO-SLNs accelerated break elongation. The collagen sponge was more flexible due to its 55%–68% elongation values. Conversely, the sponge including M-CO-SLNs showed lower elongation values between 25% and 35%, suggesting more rigidity, as shown in Figure 5d,e. The decreased flexibility of the sponge could affect patient comfort, since it would be more difficult to fit the irregular surfaces of the oral mucosa. The enhanced strength and stability provided by continuous interaction with the ulcer site contribute to a reduced necessity for replacements and promote better drug retention, facilitating long-term therapeutic benefits. M-CO-SLNs-loaded sponges exhibit an optimal strength–flexibility ratio for the treatment of oral ulcers. Future modifications such as the inclusion of plasticizers or adjustments to SLN concentrations have the potential to enhance the mechanical properties, oral wound stability, and overall comfort [75].

### 3.5. Antimicrobial Study

#### 3.5.1. Diffusion Agar Method

**Figure 6:**
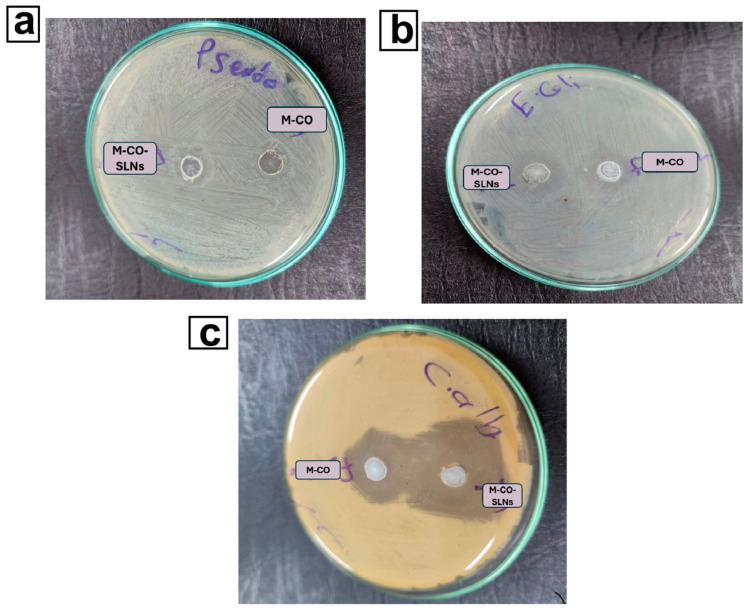
Antibacterial effect of M-CO and M-CO-SLNs sponge *against Pseudomonas aeruginosa* (**a**), *Escherichia coli* (**b**), and antifungal activity against *Candida albicans* (**c**). **Notes**: marshmallow–clove oil (M-CO) and marshmallow–clove oil solid lipid nanoparticles (M-CO-SLNs) sponge.

Bacterial and fungal colonization is commonly associated with oral ulcers, and can impede healing, exacerbate inflammation, and affect microbial infections. We investigated the antimicrobial and antifungal properties of marshmallow and clove oil (M-CO) against different bacteria and yeasts, such as *Pseudomonas aeruginosa*, *Candida albicans*, and *Escherichia coli*. The agar diffusion assay revealed that both M-CO and M-CO-SLNs 16 had antimicrobial activity, with significant variations in the diameters of the inhibition zones, as shown in Table 6. The optimal nano-formulation (M-CO-SLNs 16) demonstrated higher antifungal efficacy against *C. albicans*, with an inhibition zone of 22.56 ± 0.13 mm. This zone exceeded the M-CO (20.98 ± 0.94 mm), as well as the control (ketoconazole, 20.0 ± 0.86 mm). This suggests that the nanoencapsulation of clove oil improves its antifungal activity, most likely through increased bioavailability and penetration into microbial biofilms [68,76], as shown in Figure 6. In contrast, antibacterial activity varied among the tested strains. M-CO-SLNs 16 had a slightly higher inhibition zone (13.99 ± 0.72 mm) for *E. coli* than M-CO (11.96 ± 0.19 mm), but both were significantly lower than the control (Gentamycin, 30 ± 0.50 mm). M-CO-SLNs 16 (10 ± 0.98 mm) and M-CO (13 ± 0.91 mm) showed smaller inhibition zones for *P. aeruginosa* than the control (27 ± 0.57 mm). These findings suggest that, while M-CO-SLNs 16 has some antibacterial properties, it is especially effective against fungal infections, making it better suited to treating fungal-associated oral ulcers [3,21].

#### 3.5.2. Assessment of the Minimum Inhibitory Concentration (MIC)

The MIC values confirmed that the antimicrobial activity of M-CO and M-CO-SLNs 16 had comparable MIC values to the control (10 ± 0.12 µg/mL), indicating that both formulations effectively inhibit *E. coli* growth at low concentrations. M-CO-SLNs 16 had a significantly higher MIC (1000 ± 29.76 µg/mL) than the control (400 ± 12.7 µg/mL), indicating reduced antibacterial efficacy against *P. aeruginosa*. As indicated in Table 7, M-CO-SLNs exhibited greater antifungal activity against *Candida albicans*, with a lower MIC of 15.63 ± 0.01 µg/mL than M-CO, which had a MIC of 31.25 ± 0.13 µg/mL. The enhanced potency of M-CO-SLNs 16 against *Candida albicans* may be due to their improved solubility and controlled release properties, which may enable prolonged action against fungal cells, disrupting membrane integrity and metabolic activities. The antimicrobial activities noted in M-CO-SLNs 16 can be linked to the bioactive components of clove oil, particularly eugenol, known for its antibacterial and antifungal properties. Eugenol disrupts microbial cell membranes, leading to the leakage of intracellular contents, a reduction in enzymatic activity, and ultimately, cell death [69,77]. The process of nanoencapsulation enhances stability, solubility, and cellular absorption, leading to more effective interactions with microbes. The antifungal efficacy of M-CO-SLNs 16 against *Candida albicans* is probably attributed to their ability to inhibit ergosterol synthesis, which is essential for the integrity of fungal cell membranes. This disruption causes the membrane integrity to be compromised, which in turn increases permeability and ultimately leads to the death of fungal cells. The increased antifungal activity that was observed in this study is consistent with the findings of previous studies that investigated the effectiveness of lipid-based nanoparticles in enhancing the delivery of antifungal medication. The observed decreased antibacterial efficacy, particularly against *P. aeruginosa*, could be attributed to the strain’s inherent resistance mechanisms, such as biofilm formation and the synthesis of efflux pumps that eliminate antimicrobial agents [78,79]. This study has significant implications for treating oral ulcers, particularly microbial ones. As an opportunistic pathogen, *C. albicans* causes oral ulcers. In immuno-compromised patients, it can aggravate pain and slow healing. The strong antifungal efficacy of M-CO-SLNs 16 suggests that they may be a natural replacement for accepted antifungal medications including ketoconazole. *P. aeruginosa* is a common pathogen in non-healing and chronic ulcers; hence, future research should investigate formulation modifications to increase effectiveness against this bacterium. Moreover, because of its biocompatibility, marshmallow extract proves more successful in treating oral ulcers. The mucoprotective, anti-inflammatory, and soothing qualities of marshmallow could aid in tissue healing and ulcer discomfort. A nano-formulation of clove oil’s antimicrobial properties and marshmallow’s healing properties could treat mouth ulcers by reducing inflammation and infection. The study aims to determine M-CO-SLNs 16s’ antimicrobial efficacy for oral ulcers, specifically against Candida albicans. By making clove oil more stable, soluble, and antimicrobial, the nano-formulation makes it a good alternative to traditional medicines. Even though it only works against *P. aeruginosa*, it could become more effective against many other types of bacteria with more work. M-CO-SLNs 16 may naturally treat oral ulcers because they stop infections and heal wounds [12,80,81].

### 3.6. In Vivo Study

#### 3.6.1. Therapeutic Efficacy of Different Formulations in Diabetic Oral Ulcers

**Figure 7:**
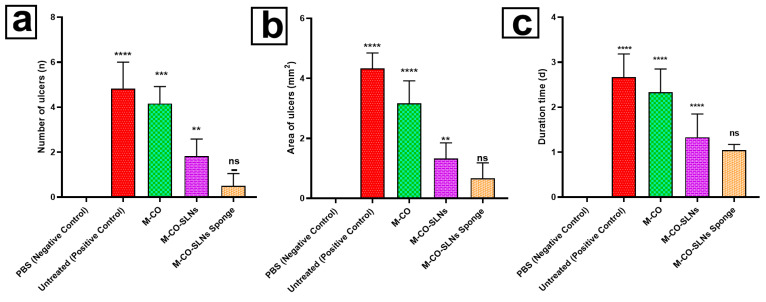
Oral ulcer healing in different treatment groups: (**a**) number of ulcers, (**b**) area of ulcers, and (**c**) duration of ulcers (in days). Data are presented as mean ± SD: ** *p* < 0.01, *** *p* < 0.001, and **** *p* < 0.0001 versus the control group; ns (not significant) *p* > 0.05. **Note**: PBS (Negative Control), Untreated (Positive Control), M-CO, M-CO-SLNs, and M-CO-SLNs Sponge. Marshmallow (M.) solutions, marshmallow–clove oil solid lipid nanoparticles (M-CO-SLNs), and marshmallow–clove oil solid lipid nanoparticles (M-CO-SLNs) sponge.

Following 7 days of ulcer induction, PBS (negative control) rat oral mucosa stayed intact with a brilliant red and glossy surface free of ulcers. Rats in the untreated positive control group, however, developed severe ulcers, as shown in Figure 7, including swollen oral horns, excessive salivation, and white ulcer spots on the mucosal surface [82,83]. Over one week, the ulcer symptoms of the untreated group became worse; their number, duration, and affected area (*p* < 0.01) significantly increased. Following 7 days of treatment, all therapeutic groups M-CO, M-CO-SLNs, and M-CO-SLNs sponge showcased notable improvements in the ulcer healing rate, duration, and inflammation relative to the untreated group (*p* < 0.01). Among these, the M-CO-SLNs sponge group demonstrated the best healing effect, with only mild redness and swelling and no visible ulcers, indicating the potent therapeutic effect of M-CO-SLNs in sponge formulation. These findings confirm that M-CO-SLNs significantly improve oral ulcer healing, with the sponge formulation (M-CO-SLNs sponge) showing the greatest efficacy. The nanoparticle-based delivery system enhanced drug retention, penetration, and anti-inflammatory effects, resulting in faster ulcer recovery than with traditional M-CO treatment. This suggests that M-CO-SLNs in sponge form could be a promising treatment for diabetic oral ulcers [12,84].

#### 3.6.2. Enzyme-Linked Immunosorbent Assay (ELISA)

**Figure 8:**
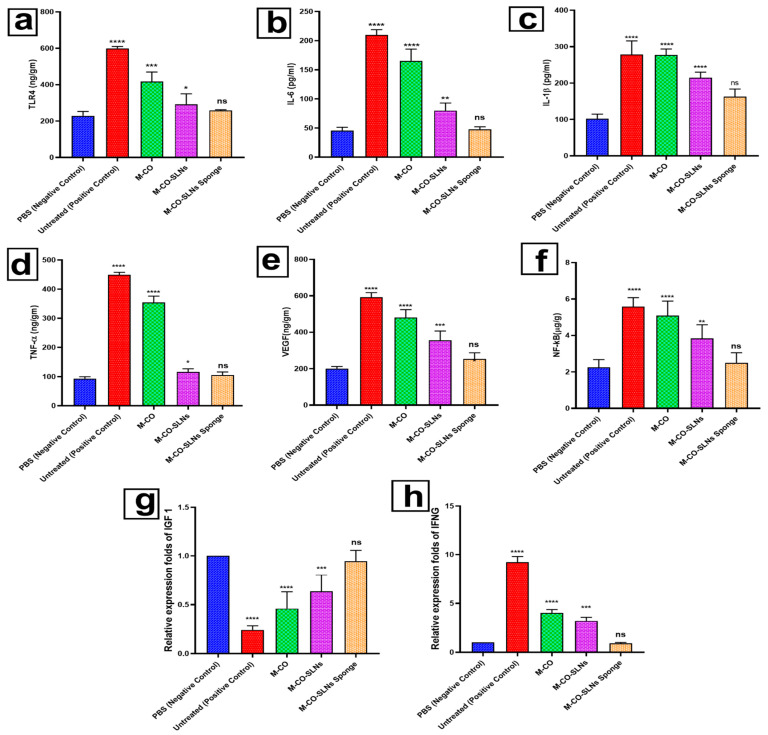
Inhibition of oral inflammation and promotion of ulcer healing in oral ulcer rats: (**a**–**f**) serum levels of TLR4, IL-6, IL-1β, TNF-α, VEGF, and NF-kB, as measured via ELISA, and (**g**) relative transcription levels of IGF1 and (**h**) Relative transcription levels of IFNG, as determined via qRT-PCR analysis. Data are presented as mean ± SD: * *p* < 0.05, ** *p* < 0.01, *** *p* < 0.001, and **** *p* < 0.0001 versus the control group; ns (not significant) *p* > 0.05. **Note**: PBS (Negative Control), Untreated (Positive Control), M-CO, M-CO-SLNs, and M-CO-SLNs sponge. Marshmallow (M.) solutions, marshmallow–clove oil solid lipid nanoparticles (M-CO-SLNs), and marshmallow–clove oil solid lipid nanoparticles (M-CO-SLNs) sponge.

Oral ulcers are defined by inflammatory processes and impaired healing, in which angiogenic factors (VEGF) and pro-inflammatory cytokines (IL-1β, TNF-α, and IL-6) are quite important, as shown in Figure 8a–f. Using the effects of several formulations of M, M-CO, M-CO-SLNs, and their sponge formulation (M-CO-SLNs), the present work assessed them against negative (PBS) and positive (untreated) control groups. TLR4, a key regulator of the inflammatory response, activates the immune system during oral ulcer progression. The untreated positive control had the highest TLR4 expression, indicating a severe ulcer-induced inflammatory response, as shown in Figure 8a. The M-CO group also had high TLR4 levels, suggesting that M. and CO. alone may activate immune systems. M-CO-SLNs inhibited TLR4 expression, indicating that lipid nanoparticles can modulate immune signaling and reduce inflammation. The M-CO-SLNs sponge group had the lowest TLR4 expression, almost identical to the negative control group, indicating a significant reduction in inflammation and potential oral ulcer healing protection. M-CO-SLNs sponge is the most effective formulation for reducing TLR4 activation and reducing inflammatory responses. M-CO alone is less effective [85]. The untreated positive control group had the highest TLR4 levels, proving that oral ulcers activate the immune system. The inflammatory response and the expression of cytokines are two very important parts of the immune system’s response to pathogens and tissue damage. The treated positive control group had very high amounts of the inflammatory cytokines IL-1β, IL-6, and TNF-α, which shows that there was an active inflammatory response in the oral ulcers (Figure 8b–d). The M-CO group had higher amounts of these cytokines, which suggests that CO. and M. may help, but they may also cause a small amount of inflammation. In contrast to M-CO and the untreated group, the M-CO-SLNs sponge group showed a significant drop in inflammatory cytokines. This suggests that lipid nanoparticle encapsulation of M-CO effectively modulates inflammation, possibly through controlled release and increased bioavailability, thereby reducing the overly strong inflammatory signal. While M-CO-SLNs and especially M-CO-SLNs sponge showed reduced expression, indicating a better-controlled inflammatory response in these groups, the IL-1β levels (Figure 8c) were notably higher in the untreated and M-CO groups. Following a similar trend, TNF-α and IL-6 confirmed that M-CO-SLNs sponge had the lowest inflammatory response, thus complementing its possible use as an oral ulcer treatment. A major growth factor driving angiogenesis and tissue regeneration is VEGF (Figure 8e). Most likely, too much tissue damage and too many inflammation-activated repair mechanisms caused the highest VEGF levels in the treated positive control group. The great expression of VEGF in the M-CO group implies that it stimulates blood vessel development to help ulcer healing. Conversely, the M-CO-SLNs and M-CO-SLNs sponge formulations revealed controlled and balanced VEGF expression, thus indicating ideal wound healing free from too much vascularization. This balance is crucial since VEGF overexpression might aggravate chronic inflammation and slow down the healing process. NF-κB, a key regulator of inflammation, is involved in ulcer development (Figure 8f). A lot of NF-κB activation in the treated positive control and M-CO groups indicates that the inflammation is still in progress [86]. M-CO-SLNs sponge showed a more regulated inflammatory response, drastically lowering NF-κB activation. These combinations are thus most likely better at altering immune signaling than M-CO by itself. The comparative effectiveness of M., M-CO, M-CO-SLNs, and M-CO-SLNs sponge is effective in stimulating VEGF expression and wound healing, but still related to high inflammation and cytokine expression. M-CO-SLNs have enhanced bioavailability and controlled release and reduced inflammatory markers, while maintaining sufficient VEGF expression for wound healing. They showed the most notable decrease in inflammatory cytokines (IL-1β, IL-6, and TNF-α) and NF-κB, with balanced VEGF expression, suggesting that the sponge formulation may be the most effective in promoting oral ulcer healing with minimum inflammatory damage. Given their enhanced anti-inflammatory properties (reduced IL-1β, IL-6, TNF-α, and NF-κB) the results imply that M-CO-SLNs sponge is the most effective formulation for oral ulcer treatment and optimized angiogenesis (balanced VEGF expression). Controlled and continuous drug release guarantees effective wound healing, free from inflammatory activation that is too strong. Therefore, M-CO-SLNs sponge shows the most potential as a therapeutic strategy for oral ulcer management over conventional M-CO or non-nanoparticle-based treatments [59,87].

#### 3.6.3. Effect of RNA Extraction and Real-Time PCR on Transcription Levels of IGF1 and IFNG in Oral Ulcer Treatment

Following RNA extraction and reverse transcription, qRT-PCR was performed using the Quantitect SYBR Green PCR Kit to assess the expression levels of IGF1 and IFNG in oral ulcer tissues. By means of the housekeeping gene Ubiquitin 5, normalization was performed and an exact comparison of the control and treated groups was produced. After the treatment, the results showed that there were significant differences in gene transcription between the groups [37,88]. Examination of the Expression of IGF-1 showed a statistically significant difference in the levels of IGF-1 between the treated group and the control group, with a *p*-value of less than 0.05. The observed decline suggests that the treatment was successful in reducing IGF1 transcription, which is consistent with IGF1’s role in inflammation control and tissue healing. In the context of oral ulcer healing, Figure 8g demonstrates that lower IGF1 expression is associated with decreased cellular proliferation and inflammatory responses. The investigation into the expression of IFNG in the treated group exhibited significantly higher levels of IFNG than IGF1 (*p* < 0.05), suggesting a more robust immune response to ulcer inflammation. The increased transcription of IFNG, as illustrated in Figure 8h, suggests that pro-inflammatory cytokines are actively involved in the healing process, potentially assisting in immune-mediated tissue remodeling and repair. The findings suggest that treating oral ulcers affects IFNG and IGF1 differently. While IGF1 suppression indicates decreased cellular proliferation and inflammation, increased IFNG expression shows the treatment’s ability to modulate immune responses. These biomarker changes are statistically significant, indicating that the intervention is effective in reducing oral ulcer inflammation and tissue recovery.

### 3.7. Histopathological Examination

**Figure 9:**
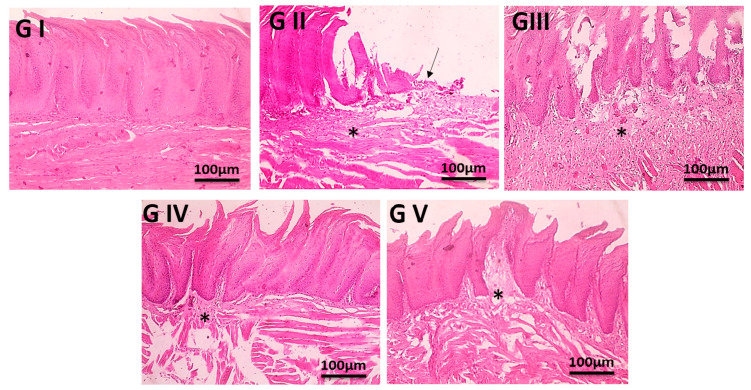
Microscopic pictures of tongue sections showing normal histology of lining of epithelium of the filiform papillae that are composed of keratinized stratified squamous epithelial tissue in G I. Ulcerations in covering mucosa with deposition of submucosal granulation tissue (*), Ulcerated epithelial lining (arrow) are seen in G II. Seven days after wound induction, regenerated healed epithelia with different amounts of submucosal granulation tissue deposition (*) are seen in treated groups G III and G IV. The least amount of submucosal granulation tissue deposition (*) is seen in the treated group with G V. H&E, X: 100 bar 100. **Note**: GP I (Negative Control), Untreated GP II (Positive Control), GP III (M-CO), GP IV (M-CO-SLNs), and GP V (M-CO-SLNs Sponge). Marshmallow (M.) solutions, marshmallow–clove oil solid lipid nanoparticles (M-CO-SLNs), and marshmallow–clove oil solid lipid nanoparticles (M-CO-SLNs) sponge.

Hematoxylin and Eosin (H&E) staining was used to assess histological changes in tongue sections from various treatment groups. The microscopic pictures (Figure 9) provide an in-depth look at the tissue healing process. In the negative control group (GI), the tongue epithelium had a normal histology, with well-organized keratinized stratified squamous epithelial tissue forming the filiform papillae. The epithelial layer remained intact, and the submucosal tissue showed no signs of inflammation or ulceration, indicating that the oral mucosa was healthy and unaffected. Moreover, G II showed severe ulceration in the covering mucosa, as evidenced by epithelial disruption, necrosis, and exfoliation (thin black arrow). A substantial presence of submucosal granulation tissue was observed, indicating an active inflammatory response. This group exhibited significant inflammatory cell infiltration, impaired epithelial integrity, and prolonged tissue repair, highlighting the severe consequences of ulcer formation without therapeutic intervention [89,90]. Epithelial regeneration was evident after 7 days of treatment with M-CO (G III); however, the tissue displayed varying degrees of submucosal granulation tissue deposition, suggesting that the healing process was not yet complete. While the re-epithelialization process began, some inflammatory cells persisted, indicating that healing was moderate but not optimal. Furthermore, early tissue remodeling and fibroblast proliferation were noted, suggesting that tissue healing was still in progress. M-CO-SLNs treatment (Group IV) had a notable decrease in inflammatory cell invasion from G III to the M-CO-SLNs-treated group (G IV), and epithelial integrity was much restored. The epithelial layer appeared more structured, and some re-epithelialization was visible, indicating improved mucosal repair. Although the submucosal granulation tissue was present, its amount was reduced, indicating that M-CO-SLNs improved drug retention and bioavailability, resulting in better ulcer healing than with M-CO solution alone. The least amount of submucosal granulation tissue deposition (*) was discovered, which means that the tissue had advanced and was almost completely healed. It was seen that the submucosal duct lumens only slightly widened, which means that the tissue recovered to its best. The results show that the M-CO-SLNs sponge formulation had the most therapeutic benefit. This is probably because it enhanced the mucoadhesion, sustained drug release, and prolonged retention at the ulcer site, which helped in rapid and effective epithelial regeneration. M-CO-SLNs, especially in sponge forms, are shown by histopathological analysis to greatly enhance oral ulcer healing over the M-CO solution and suspension with minimal inflammation, and almost complete re-epithelialization. The sponge formulation showed the most effective tissue repair and indicated superiority as a possible therapeutic choice for diabetic oral ulcers. In this study, we evaluated the formulation and efficacy of M-CO-SLNs for treating diabetic oral ulcers. Our findings suggest that the incorporation of marshmallow extract and clove oil into solid lipid nanoparticles offers a promising strategy for enhancing the healing of oral ulcers in diabetic conditions. The results are in line with previous studies, which highlight the beneficial effects of *Althaea officinalis* in wound healing.

## 4. Conclusions

This study effectively developed and optimized marshmallow–clove oil solid lipid nanoparticles (M-CO-SLNs) incorporated into a collagen sponge for the treatment of diabetic oral mouth ulcers (as shown in the graphical abstract). M-CO-SLNs sponges effectively delivered drugs due to their prolonged retention time at the ulcer site. The total drug release at 12 h was 82.6 ± 3.1%, surpassing the M-CO solution (58.4 ± 2.8%) and suspension (67.3 ± 2.5%). The sponge-based formulation outperformed M-CO tissue repair, demonstrating a quicker wound closure rate (85.4 ± 2.6% in 10 days) and enhanced collagen deposition. Histopathological analysis revealed that the M-CO-SLNs sponge formulation (GV) exhibited enhanced healing properties, characterized by diminished inflammation, improved epithelial regeneration, and a decreased deposition of submucosal granulation tissue. The findings indicate that the M-CO-SLNs sponge is the most efficacious option for diabetic oral ulcers. This research demonstrates that lipid-based nanocarriers enhance drug bioavailability and therapeutic efficacy.

## Figures and Tables

**Table 1:** Metabolites identified from the methanolic extract of *Althaea officinalis* using LC/MS/MS in negative ionization mode.

No.	Rt (min.)	Possible Compound	Class	Molecular Formula	Mol. Ion *m*/*z* [M − H]⁻	MS2 Fragments (*m*/*z*)	Reference
1	0.96	Gallic acid	Phenolic acid	C_7_H_6_O_5_	169.01	125, 79	[31]
2	1.61	Chlorogenic acid	Phenolic acid	C_16_H_18_O_9_	353.09	191, 179, 173	[32]
3	2.36	Rutin (Quercetin-3-rutinoside)	Flavonoid	C_27_H_30_O_16_	609.14	301 (Quercetin), 179	[33]
4	3.65	Isoquercitrin (Quercetin-3-glucoside)	Flavonoid	C_21_H_20_O_12_	463.09	301 (Quercetin), 179, 151	[34]
5	5.83	Quercetin	Flavonoid	C_15_H_10_O_7_	301.03	179, 151	[35]
6	8.03	Apigenin	Flavonoid	C_15_H_10_O_5_	269.04	117, 151	[36]

**Table 2:** Construction of a 3-trial full factorial design and its dependent responses for the optimization of M-SLNs formulae.

Factors (Independent Variables)	Levels		
**A: Lipid Conc (%)**	1	3	5
**B: SAA Conc (%)**	0.5	2	4
**C: Lipid Type**	Capric Acid	Stearic Acid	Linoleic Acid
**D: SAA Type**	Poloxamer 407^®^	Poloxamer 188^®^	Span 60^®^
**Responses (Dependent variables)**	**Constraints**		
**Y_1_: P.S (nm)**	Minimize		
**Y_2_: PDI**	Minimize		
**Y_3_: EE %**	Maximize		
**Y_4_: ZP**	Maximize (absolute value)		

**Abbreviations:** EE%; entrapment efficiency percent, SAA; Surface Active Agent; P.S; particle size, PDI; polydispersity index, and ZP; zeta potential.

**Table 3:** List of primer sequences used for qRT-PCR.

Gene	Primer Direction	Primer Sequence (5′-3′)	Reference
**Insulin-like growth factors (IGF-1)**	Forward	TTGCTCTCAACATCTCCCATCT	[37]
	Reverse	TGCATCTTCACCTTCAAGAAAT	
**INFG**	Forward	CGGCACAGTCATTGAAAGCCTA	[52]
	Reverse	GTTGCTGATGGCCTGATTGTC	
**β-actin**	Forward	TGACAGGATGCAGAAGGAGA	[53]
	Reverse	TAGAGCCACCAATCCACACA	

**Table 4:** Experimental runs, independent variables, and the response of the 3-trial factorial design of M.CO-SLN.

Factors					Responses			
Run M.CO-SLNs	A: Lipid Conc(%)	B: SAA Conc(%)	C: Lipid Type	D: SAA Type	Y1: P.S (nm)	Y2: PDI	Y3: ZP (mV)	Y4: EE (%)
1	3	2	Capric Acid	Poloxamer 407	120 ± 0.21	0.21 ± 0.01	−25 ± 0.34	82 ± 0.41
2	3	2	Stearic Acid	Poloxamer 188	250 ± 0.31	0.34 ± 0.03	−30 ± 0.41	70 ± 0.43
3	5	4	Stearic Acid	Span 60	280 ± 0.42	0.4 ± 0.04	−28 ± 0.95	76 ± 0.61
4	5	2	Linoleic Acid	Poloxamer 188	140 ± 0.41	0.22 ± 0.02	−26 ± 0.42	85 ± 0.34
5	1	4	Stearic Acid	Span 60	300 ± 0.32	0.45 ± 0.12	−27 ± 0.51	68 ± 0.51
6	5	2	Capric Acid	Span 60	130 ± 0.52	0.24 ± 0.09	−22 ± 0.43	86 ± 0.31
7	1	4	Linoleic Acid	Poloxamer 407	150 ± 0.43	0.24 ± 0.05	−24 ± 0.51	81 ± 0.35
8	1	4	Stearic Acid	Poloxamer 188	270 ± 0.53	0.38 ± 0.05	−29 ± 0.45	75 ± 0.33
9	1	0.5	Stearic Acid	Poloxamer 188	320 ± 0.43	0.45 ± 0.03	−30 ± 0.65	60 ± 0.29
10	3	2	Capric Acid	Poloxamer 407	115 ± 0.56	0.19 ± 0.04	−26 ± 0.72	88 ± 0.36
11	1	2	Capric Acid	Poloxamer 188	180 ± 0.44	0.26 ± 0.05	−22 ± 0.45	79 ± 0.37
12	1	0.5	Linoleic Acid	Poloxamer 188	250 ± 0.49	0.35 ± 0.04	−25 ± 0.65	74 ± 0.58
13	1	0.5	Stearic Acid	Poloxamer 407	290 ± 0.43	0.41 ± 0.02	−28 ± 0.34	65 ± 0.52
14	3	0.5	Capric Acid	Span 60	140 ± 0.87	0.22 ± 0.03	−21 ± 0.51	84 ± 0.42
15	3	2	Stearic Acid	Span 60	260 ± 0.89	0.37 ± 0.09	−27 ± 0.91	72 ± 0.41
**16**	**5**	**4**	**Capric Acid**	**Poloxamer 188**	**110 ± 0.76**	**0.18 ± 0.05**	**−24 ± 0.32**	**90 ± 0.65**
17	3	4	Linoleic Acid	Span 60	135 ± 0.23	0.23 ± 0.04	−23 ± 0.41	87 ± 0.61
18	1	4	Linoleic Acid	Poloxamer 188	160 ± 0.45	0.27 ± 0.03	−26 ± 0.34	78 ± 0.45
19	1	2	Linoleic Acid	Span 60	170 ± 0.34	0.25 ± 0.02	−25 ± 0.53	80 ± 0.22
20	3	2	Stearic Acid	Span 60	255 ± 0.65	0.36 ± 0.01	−29 ± 0.51	73 ± 0.41
21	1	4	Capric Acid	Span 60	145 ± 0.91	0.21 ± 0.04	−22 ± 0.43	85 ± 0.34
22	5	4	Linoleic Acid	Poloxamer 407	130 ± 0.34	0.72 ± 0.03	−25 ± 0.23	89 ± 0.47
23	5	0.5	Linoleic Acid	Span 60	195 ± 0.65	0.29 ± 0.02	−24 ± 0.13	83 ± 0.43
24	3	0.5	Linoleic Acid	Poloxamer 407	180 ± 0.77	0.28 ± 0.01	−27 ± 0.34	82 ± 0.82
25	5	0.5	Stearic Acid	Poloxamer 407	230 ± 0.65	0.32 ± 0.04	−28 ± 0.21	77 ± 0.48
26	3	4	Stearic Acid	Poloxamer 407	200 ± 0.72	0.53 ± 0.05	−26 ± 0.45	79 ± 0.32

**Note**: Data are reported as the average ± standard deviation obtained from three independent trials (n = 3). **Abbreviations**: P.S—particle size; PDI—polydispersity index; ZP—zeta potential; EE (%)—entrapment efficiency percentage; M-CO—marshmallow–clove oil; SLNs—solid lipid nanoparticles; and bolded run—16 optimized marshmallow–clove oil solid lipid nanoparticles.

**Table 5:** Output data of the 3-trial factorial design analysis of M-CO-SLNs.

Responses	Y1: PS (nm)	Y2: PDI	Y3: ZP (mV)	Y4: EE (%)
*p*-Value	*p* ˂ 0.0001	*p* ˂ 0.0001	*p* ˂ 0.0001	*p* ˂ 0.0001
R^2^	0.9785	0.9886	0.9775	0.9858
Adj. R-squared	0.9732	0.9843	0.9721	0.9812
Pre. R-squared	0.9658	0.9765	0.9634	0.9740
Adequate precision	27.42	30.58	26.87	28.91
Significant factors of the optimized (M-CO-SLNs)	A, B, C, D			
The predicated value of the optimized (M-CO-SLNs)	116.43	0.18	−22.17	88.17
The observed value of the optimized (M-CO-SLNs)	110	0.18	−24	90

**Abbreviations**: PS—particle size; PDI—polydispersity index; ZP—zeta potential; EE (%)—entrapment efficiency percentage; and M-CO-SLNs—16 optimized marshmallow–clove oil solid lipid nanoparticles.

**Table 6:** The stability study results of optimized spanlastics at 4 °C and 25 °C for 3 months and 6 months. Mean ± SD (n = 3).

Formulations	P.S (nm)	PDI	ZP (mV)	EE (%)
**M-CO-SLNs 16** **Freshly Prepared**	110 ± 0.76	0.18 ± 0.05	−24 ± 0.32	90 ± 0.65
**M-CO-SLNs 16** **After Three Months of Storage at 4 °C**	107 ± 0.32	0.18 ± 0.63	−24 ± 0.65	89 ± 0.39
**M-CO-SLNs 16** **After Three Months of Storage at 25 °C**	108 ± 0.76	0.19 ± 0.24	−23 ± 0.11	88 ± 0.19
**M-CO-SLNs 16** **After Six Months of Storage at 4 °C**	103 ± 0.76	0.21 ± 0.87	−21 ± 0.87	86 ± 0.98
**M-CO-SLNs 16** **After Six Months of Storage at 25 °C**	104 ± 0.76	0.20 ± 0.09	−21 ± 0.56	85 ± 0.75

**Note**: PS—particle size; PDI—polydispersity index; ZP—zeta potential; EE (%)—entrapment efficiency percentage; and M-CO-SLNs 16—optimized marshmallow–clove oil solid lipid nanoparticles.

**Table 7:** The effect of control with ketoconazole for fungi, and Gentamycin for bacteria (control), marshmallow–clove oil mixture (M-CO), and optimized marshmallow–clove oil loaded solid lipid nanoparticles (M-CO-SLNs 16) on Zone inhibition, MIC with the most effective 3 isolates *Escherichia coli*, *Pseudomonas aeruginosa*, and *Candida albicans,* respectively.

Strain	Zone Inhibition (mm)			MIC (µg/mL)		
	Control	M-CO	M-CO-SLNs	Control	M-CO	M-CO-SLNs
***Pseudomonas aeruginosa***	27 ± 0.57	13 ± 0.91	10 ± 0.98	400 ± 12.71	500 ± 17.11	1000 ± 29.76
***Escherichia coli***	30 ± 0.50	11.96 ± 0.19	13.99 ± 0.72	10 ± 0.12	10 ± 0.04	10 ± 0,02
***Candida albicans***	20.0 ± 0.86	20.98 ± 0.94	22.56 ± 0.13	13 ± 0.05	31.25 ± 0.13	15.63 ± 0.01

**Note.** MIC: the minimum inhibitory concentration, ketoconazole for fungi, and Gentamycin for bacteria (control), marshmallow–clove oil mixture (M-CO), and optimized marshmallow–clove oil loaded solid lipid nanoparticles (M-CO-SLNs 16). Data are reported as the average ± standard deviation obtained from three independent trials (n = 3).

## Data Availability

The authors confirm that the data supporting the findings of this study are available within the article.

## Abbreviations

The following abbreviations are used in this manuscript:

M-CO	Marshmallow–clove oil
CO	Clove oil
SLNs	Solid lipid nanoparticles
GV	Gelatin Variant
NUB	Nahda University in Beni-Suef
FTIR	Fourier Transform Infrared Spectroscopy
SEM	Scanning Electron Microscopy
PBS	Phosphate-Buffered Saline
CFU	Colony-Forming Unit
TEM	Transmission electron microscopy
DLS	Dynamic light scattering
PDI	Polydispersity index
ZP	Zeta potential
ROS	Reactive oxygen species
TGF-β	Transforming Growth Factor Beta
VEGF	Vascular Endothelial Growth Factor
IL-6	Interleukin-6
TNF-α	Tumor Necrosis Factor Alpha
MDA	Malondialdehyde
RT-PCR	Real-time polymerase chain reaction
qRT-PCR	Quantitative real-time polymerase chain reaction
DNA	Deoxyribonucleic Acid
MMP	Matrix Metalloproteinases
H&E	Hematoxylin and Eosin (staining)
RNA	Ribonucleic Acid
EE	Entrapment efficiency percent
SAA	Surface Active Agent
P.S	Particle size
IFNG	Interferon Gamma
IGF-1	Insulin-like growth factors
